# The importance of intravenous glucose tolerance test glucose stimulus for the evaluation of insulin secretion

**DOI:** 10.1038/s41598-024-54584-x

**Published:** 2024-03-28

**Authors:** Ian F. Godsland, Desmond G. Johnston, KGMM Alberti, Nick Oliver

**Affiliations:** 1 Division of Metabolism, Digestion and Reproduction, Faculty of Medicine, Imperial College London, London, UK; 2 Wynn Reader in Human Metabolism, Section of Metabolic Medicine, Faculty of Medicine, Imperial College London (St Mary’s Campus), Room G1, Norfolk Place, London, W2 2NH UK; 3 Department of Diabetes and Endocrinology, St Marys Hospital, Imperial College Healthcare NHS Trust, London, UK

**Keywords:** Endocrinology, Medical research

## Abstract

For 100 years, the Intravenous glucose tolerance test (IVGTT) has been used extensively in researching the pathophysiology of diabetes mellitus and AIRg—the IVGTT-induced acute insulin response to the rapid rise in circulating glucose—is a key measure of insulin secretory capacity. For an effective evaluation of AIRg, IVGTT glucose loading should be adjusted for glucose distribution volume (gVOL) to provide an invariant, trend-free immediate rise in circulating glucose (ΔG0). Body weight-based glucose loads have been widely used but whether these achieve a trend-free ΔG0 does not appear to have been investigated. By analysing variation in AIRg, ΔG0 and gVOL with a range of IVGTT loads, both observed and simulated, we explored the hypothesis that there would be an optimum anthropometry-based IVGTT load calculation that, by achieving a trend-free ΔG0, would not compromise evaluation of AIRg as an index of beta cell function. Data derived from patient and research volunteer records for 3806 IVGTT glucose and insulin profiles. Among the non-obese, as gVOL rose, weight increased disproportionately rapidly. Consequently, the IVGTT glucose load needed for an invariant ΔG0 was progressively overestimated, accounting for 47% of variation in AIRg. Among the obese, ΔG0 was trend-free yet AIRg increased by 11.6% per unit body mass index, consistent with a more proportionate increase in weight with gVOL and a hyperinsulinaemic adaptation to adiposity-associated insulin resistance. Simulations further confirmed our hypothesis by demonstrating that a body surface area-based IVGTT load calculation could provide for a more generally invariant IVGTT ΔG0.

## Introduction

In research into the pathophysiology of diabetes mellitus, intravenous injection of glucose was first described by Jørgensen and Plum in 1923^1^. In the 100 years since this report, developments have included the introduction of IVGTT glucose loads calculated to correct for variation in distribution volume^2,3^ and the use of a mono-exponential fit to the glucose concentration decay profile to provide a diagnostic index for diabetes^4,5^. Most important, however, has been the combining of insulin sensitivity (Si) measurements derived using the Bergman/Cobelli minimal model of glucose disappearance^6^ with the area under the IVGTT insulin concentration profile in the first 10 min of the IVGTT—the first-phase, acute insulin response to glucose (AIRg). The combination of these two measures then synergised to transform the IVGTT into a versatile research tool capable of providing measures of both insulin sensitivity and secretion from a single investigative procedure^7,8^ that continues in current use^9,10^.

Insulin resistance (i.e., low Si), is widely recognised as a factor in metabolic disturbances associated with risks of diabetes and cardiovascular disease and a decrease in AIRg is one of the first signs of failure of the beta cells of the pancreas to mount an adequate insulin secretion response to a rise glucose concentrations^11^. Moreover, low AIRg is an independent predictor of incident diabetes^12^. Consequently, the combination of Si and AIRg has been widely used in clinical research studies and these measures are now well-characterised. However, very little attention has been given to any influence there may be of IVGTT glucose loading on IVGTT-derived measures. It has been assumed that loading calculated in proportion to body weight will correct for variation in glucose distribution volume and, in so doing, generate an immediate rise in glucose concentrations, ΔG0, that, as the stimulus for AIRg, shows no trend with variation in body size that might bias measurement of AIRg. However, whether weight-based glucose loading has succeeded in ensuring the invariant ΔG0 required for unbiased evaluation of AIRg across a range of body sizes does not appear to have been confirmed.

Rather than being dependent on absolute glucose concentrations, minimal model measurement of Si utilises rates of change in glucose concentrations and their relationships with the accompanying insulin concentrations. However, this is not the case for AIRg, which may vary with the absolute glucose concentrations. Consequently, for weight-based glucose loading, if weight does not change proportionally with the glucose distribution volume, gVOL, the immediate glucose stimulus to insulin secretion generated by the glucose injection may not be strictly invariant between individuals and spurious associations may be generated between variables related to insulin secretion and variables related to weight, for example adiposity.

By analysing variation in AIRg, ΔG0 and gVOL with a range of IVGTT loads, both observed and simulated, we explored the hypothesis that there would be an optimum anthropometry-based IVGTT load calculation that, by achieving a trend-free ΔG0, would not compromise evaluation of AIRg as an index of beta cell function. A comprehensive analysis of associations has been made possible by the 3,806 IVGTT glucose and insulin concentration records included in a substantial body of metabolic data, the Wynn Database. Trends in ΔG0 generated by disproportionate variation with gVOL in weight and other anthropometric measures that may be used to calculate IVGTT glucose loads have been identified. This has then enabled quantitative estimation of the influence of variation with gVOL on associations of AIRg with adiposity. Both observed and simulated data have been analysed to clarify these relationships and IVGTT loads calculated according to a range of IVGTT loading formulae have been assessed for their ability to achieve variation in the immediate rise in glucose concentrations free of trend in relation to anthropometry.

## Results

After application of inclusion criteria (see Methods), the present analysis was restricted to 3,806 IVGTT glucose and insulin concentration records for 2434 participants. Evaluation of records meeting inclusion criteria then indicated that two distinct IVGTT datasets could be usefully distinguished:

1. the ‘IG16’ dataset, characterised by IVGTTs with plasma glucose and insulin concentrations recorded over 180 min and with 16 samples taken, including two fasting samples plus the samples necessary for calculating AIRg. There were 2,951 IG16 dataset IVGTTs carried out for 1,929 participants and the majority of glucose loads given were calculated according to 0.5 g per kg body weight. However, to diminish the injected glucose burden for obese participants, an IVGTT glucose load calculation of 20 g per m^2^ body surface area (BSA) could be employed and this BSA-based load was recorded as having been given to 249 of the 369 obese participants represented in the Database. Obesity was defined as a weight ≥ 120% of ideal body weight (%IBW), with ideal body weights derived from the actuarial tables used by the Metropolitan Life Insurance Company to assess health risks in insured individuals^13^. Percent IBW was adopted for evaluation of adiposity for Wynn Database participants from the outset of data recording in 1965, which was prior to the widespread use of body mass index (BMI) for the same purpose. Nevertheless, among Wynn Database IVGTTs, the correlation coefficient between %IBW and BMI was 0.985 and, based on a regression analysis, 120%IBW was found to be equivalent to a BMI of 27.5 kg/m^2^ indicating a very close relationship between the two indices.
2. the ‘IG9’ dataset was characterised by plasma glucose and insulin concentrations recorded over 90 min and with 9 samples taken. There were 855 IG9 dataset IVGTTs carried out for 499 participants, with 0.5 g per kg body weight IVGTT loading for all participants, regardless of adiposity. Very few IG9 dataset records included the samples necessary for calculating AIRg but the IG9 records were, nevertheless, suitable for investigating associations between IVGTT glucose load and ΔG0.

In the principal IG16 dataset, ages ranged between 19 and 82 years, 13% of participants were obese and substantial proportions of participants had diagnoses and/or were taking medications likely to affect carbohydrate metabolism (Table 1). Among IG9 dataset participants, there was a significantly higher proportion of women than in the IG16 set (88 v 61%, *p <* 0.001), a younger median age (31.0 vs. 53.1 years, *p <* 0.001) and a higher incidence of obesity (24 vs. 13%, *p <* 0.001), although there appeared to be less adiposity, overall, as judged by the lower median BSA, %IBW, BMI and calculated fat-free mass. IG9 set participants also had a greater frequency of diagnoses and medication use likely to affect carbohydrate metabolism (30 vs. 15%, *p <* 0.001). Median values for ΔG0 were 15.4 mmol/L and 15.5 mmol/L (*p* = 0.08, Table 1) in IG9 and IG16 sets, respectively and median calculated glucose distribution volumes, gVOL, were 11.5 and 12.1L (*p <* 0.001). The IG9 set also exhibited lower insulin sensitivity (0.35 vs. 0.51 min^−1^, *p <* 0.001). Only 30 IG9 participants had insulin measurements for calculating AIRg but their median AIRg was, nevertheless, very close to the median for the IG16 dataset (3195 vs. 3160 pmol/L min). Of the 2,951 IVGTTs included in the IG16 set, 2566 had accompanying C-peptide concentrations recorded, which could provide an indication as to whether variation in insulin concentrations was due to variation in insulin secretion or insulin elimination^14^.

**Table 1:** IVGTT set characteristics. (Man-Whitney or chi square significances for the difference between IG9 and IG16 sets are shown in superscript against the the IG9 set values).

	IG16 set (n = 2,951 IVGTTs for 1,929 participants)	IG9 set (n = 855 IVGTTs for 499 participants)
women, % (n)	61 (1803)	88 (751)^<0.001^
Age (years), median (range. IG9 n = 645)	53.1 (18.9, 82.2)	31.0 (12.8, 65.5)^<0.001^
Weight ≥ 120%IBW % (n)	13 (369)	24 (202)^<0.001^
Excluded diagnoses/medications % (n)		
none	31 (1020)	19 (160)^<0.001^
excluded diagnoses	37 (1088)	54 (465)^<0.001^
excluded medications	44 1289)	57 (489)^<0.001^
excluded diagnoses and medications	15 (445)	30 (258)^<0.001^
Anthropometry, median (range)		
weight (kg)	67.7 (39.5, 133.0)	62.5 (31.6, 109.6)^<0.001^
body surface area (m^2^)	1.77 (1.30, 2.61)	1.70 (1.15, 2.39)^<0.001^
% ideal body weight	106 (68, 192)	103 (59, 197)^0.03^
body mass index (kg/m^2^)	24.2 (15.8, 43.0)	23.1 (13.9, 45.3)^<0.001^
fat-free mass (kg, IG9 n = 645)	45.8 (31.0, 88.5)	43.9 (32.7, 79.0)^<0.001^
height (cm)	167 (145, 197)	163 (142, 200)^<0.001^
IVGTT. median (range)		
mean fasting glucose (mmol/L)	5.08 (3.81, 6.95)	4.75 (2.78, 6.97)^<0.001^
glucose load (g)	33.9 (19.8, 52.5)	31.3 (15.8, 54.8)^<0.001^
ΔG0 (mmol/L)	15.5 (8.2, 22.9)	15.4 (7.9, 27.2)^0.08^
gVOL (L)	12.1 (6.8, 20.0)	11.5 (4.3, 20.4)^<0.001^
AIRg (pmol/L.min, IG9 n = 30)	3160 (11, 26,109)	3195 (114, 15,502)^0.8^
Si (/min/pmol/L)	0.51 (0.05, 4.38)	0.35 (0.02, 3.45)^<0.001^
Sg (/min)	1.56 (0.24, 6.31)	1.91 (0.33, 6.74)^<0.001^

### Variation with adiposity in the difference in IVGTT load given according to 0.5 g/kg or 20 g/m^2^ IVGTT glucose loading

The BSA-based IVGTT load was originally introduced to reduce the IVGTT glucose injection burden in the obese, but the extent to which this succeeded does not appear to have been reported. In a preliminary investigation in the principal IG16 dataset, we determined to what extent, as adiposity increased, BSA-based IVGTT loading reduced glucose loading relative to weight-based loading. For each IVGTT, the difference in load between a BSA-based IVGTT load and a weight-based IVGTT load were plotted against %IBW, %IBW being the measure used to discriminate obesity and use of 20g/m^2^ IVGTT loading.

**Figure 1:**
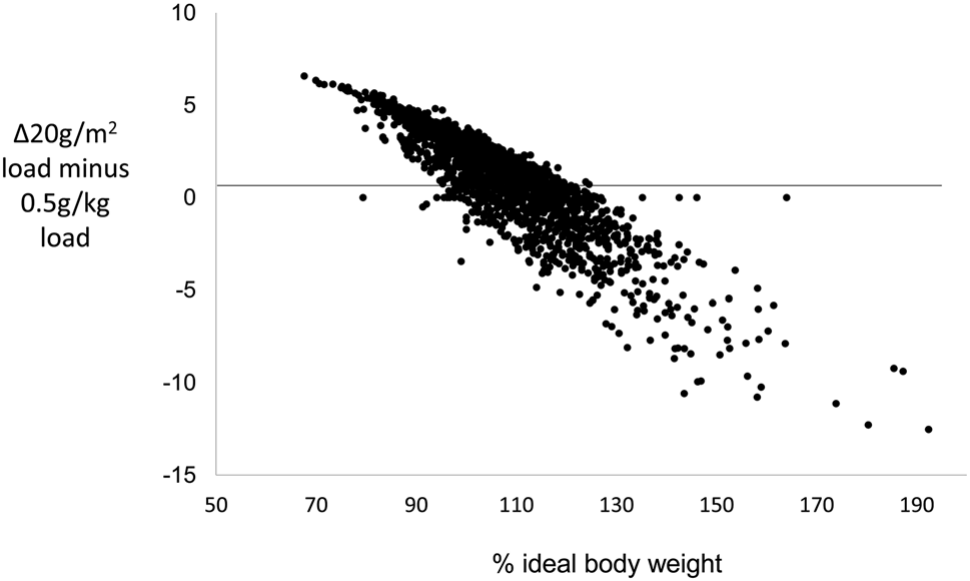
Differences in IVGTT loads calculated according to 20 g/m^2^ body surface area or 0.5 g/kg body weight. Differences are plotted relative to %IBW in the IG16 dataset (n = 2951).

At low %IBW the 20g/m^2^ calculation generated a higher load than the 0.5g/kg calculation, whereas at high %IBW it generated a lower load, resulting in a continuous negative relationship between the differences in loads and %IBW (Fig. 1). Regression analysis of %IBW as a predictor of the difference in loads indicated zero difference between loads at 114%IBW. Overall, differences between loads ranged from + 6.6 to − 12.5 g. The trend in loading difference suggested that the two loadings would be expected to differ in the extent to which they could sustain an invariant ΔG0 across the full range of adiposity.

### Qualitative trends with adiposity in the immediate rise in IVGTT glucose concentration, ΔG0, according to IVGTT load, obesity and dataset

**Figure 2:**
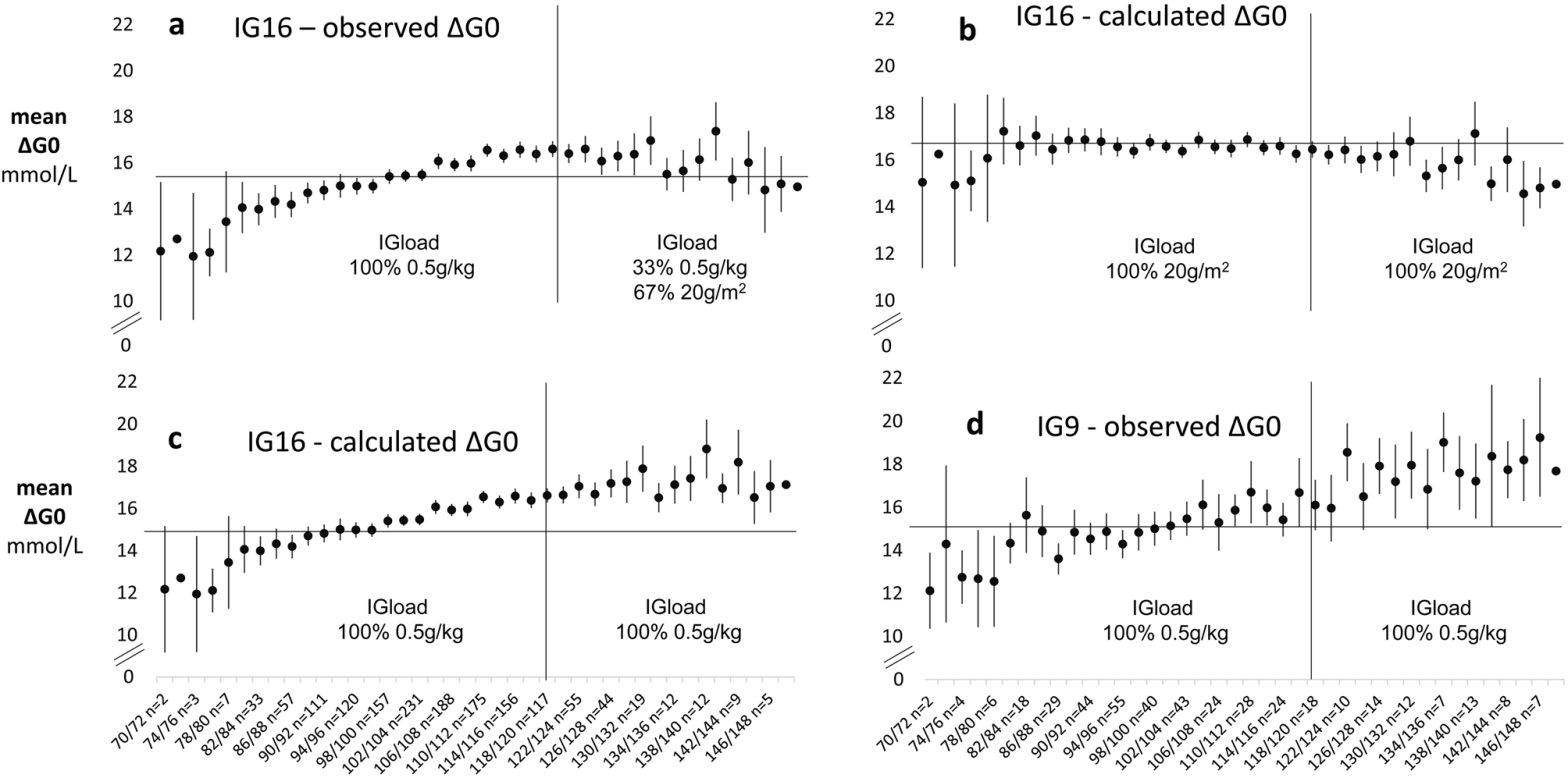
Variation in the immediate rise in IVGTT glucose concentrations, ΔG0, with percentage of ideal body weight (%IBW) as a measure of adiposity. Mean (95%CI) observed and calculated values for ΔG0 are shown in 2% strata of %IBW in IG16 (n = 2951 IVGTTs) and IG9 (n = 855 IVGTTs) datasets. Calculated values of ΔG0 are those that would have obtained had 20 g/m^2^ body surface area been used for IVGTTs carried out using 0.5 g/kg or 20 g/m^2^ body surface area been used for IVGTTs carried out using the 0.5 g/kg loading calculation. Calculated values were derived for each IVGTT by dividing by the measured glucose distribution volume the IVGTT load that would have been given had either 0.5 g/kg body weight or 20 g/m^2^ body surface area load been used. The x-axis indicates the range of %IBW in each stratum and is shown with the numbers of observations in each stratum. NOTE—y-axes have been truncated to clarify overall trends.

Variation in ΔG0 with adiposity and IVGTT load was visualised by plotting profiles for mean ΔG0 in 2% strata of %IBW between 70 and 150%IBW (outside these limits an increasing number of strata had no observations).At the extremes of %IBW, numbers were small and confidence intervals wide. Nevertheless, mean values for ΔG0 in the IG16 set could be seen to rise steadily with increasing %IBW to about 120%IBW, after which there was little evidence of any trend (Fig. 2a).

The absence of a continuing positive trend in ΔG0 above 120%IBW in the IG16 set was consistent with the progressively lower IVGTT loading generated by the admixture of 20g/m^2^ load-calculated IVGTTs among the obese. To investigate this possibility further, we first calculated the IVGTT loads that would have obtained for 20g/m^2^ loading in participants who had received a 0.5g/kg load and for 0.5g/kg loading among those who had received a 20g/m^2^ load. Values of ΔG0 were then calculated using measured values for gVOL in the formula: ΔG0_calculated_ = IVGTT load _(20g/m2 or 0.5g/kg)_/gVOL_measured_. Mean values of ΔG0_observed_ were then combined with ΔG0_calculated_ in 2% strata of %IBW to provide ΔG0 profiles for the IG16 participants that would have obtained had they received exclusively 20g/m^2^ or exclusively 0.5g/kg loads across the full range of %IBW (Figs. 2b,c, respectively). With 20g/m^2^ loading throughout, ΔG0 showed relatively little trend in the non-obese and a slight negative trend in those ≥ 120%IBW. With 0.5g/kg loading throughout, ΔG0 continued to increase beyond 120%IBW at a similar rate to that seen in non-obese participants. The continuing rise in ΔG0 with 0.5g/kg loading throughout was confirmed by the observed mean ΔG0 profile in the exclusively 0.5g/kg-loaded IG9 set, (Fig. 2d). The observed ΔG0 profile for the IG16 set (Fig. 2a) was therefore consistent with the 67% admixture of 20g/m^2^ loaded IVGTTs in those ≥ 120%IBW.

### Quantitative trends with adiposity and other measures of body size in the immediate rise in IVGTT glucose concentration, ΔG0, according to IVGTT glucose loading, obesity and dataset

The trends in ΔG0 with %IBW visualised in the previous section were quantified by regression analysis and the investigation was extended to include not only associations with %IBW but also with weight, BSA, body mass index (BMI), fat-free mass (FFM) and height as alternative measures of body size. These measures were entered as regression analysis predictors as standardised values to enable between-measure comparisons in their strengths of association with ΔG0. Associations of ΔG0 with each of the 6 measures were assessed according to IVGTT loading (0.5 g/kg and 20 g/m^2^), obesity (< 120%IBW and ≥ 120%IBW) and dataset (IG9 and IG16). Accordingly, for each of the 6 anthropometric measures, 5 groups of observations were distinguished:

- IG9 set, <120%IBW, receiving 0.5g/kg, n=652
- IG16 set, <120%IBW, receiving 0.5g/kg, n=2547
- IG9 set, ≥120%IBW, receiving 0.5g/kg, n=202
- IG16 set, ≥120%IBW, receiving 0.5g/kg, n=120
- IG16 set, ≥120%IBW, receiving 20g/m^2^, n=249

By calculating, as described in the previous section, the ΔG0 that would have obtained in Group e had a 0.5 g/kg load been given and in Groups a–d had a 20 g/m^2^ load been given, 2 sets of the 5 groups were then generated—one for 0.5 g/kg loading in each of the 5 groups and the other for 20 g/m^2^ loading. Regression coefficients for associations of ΔG0 with each of the 6 anthropometric measures were then derived in each of the 5 groups for each of the 2 IVGTT loads (Supplementary Table 1).

**Figure 3:**
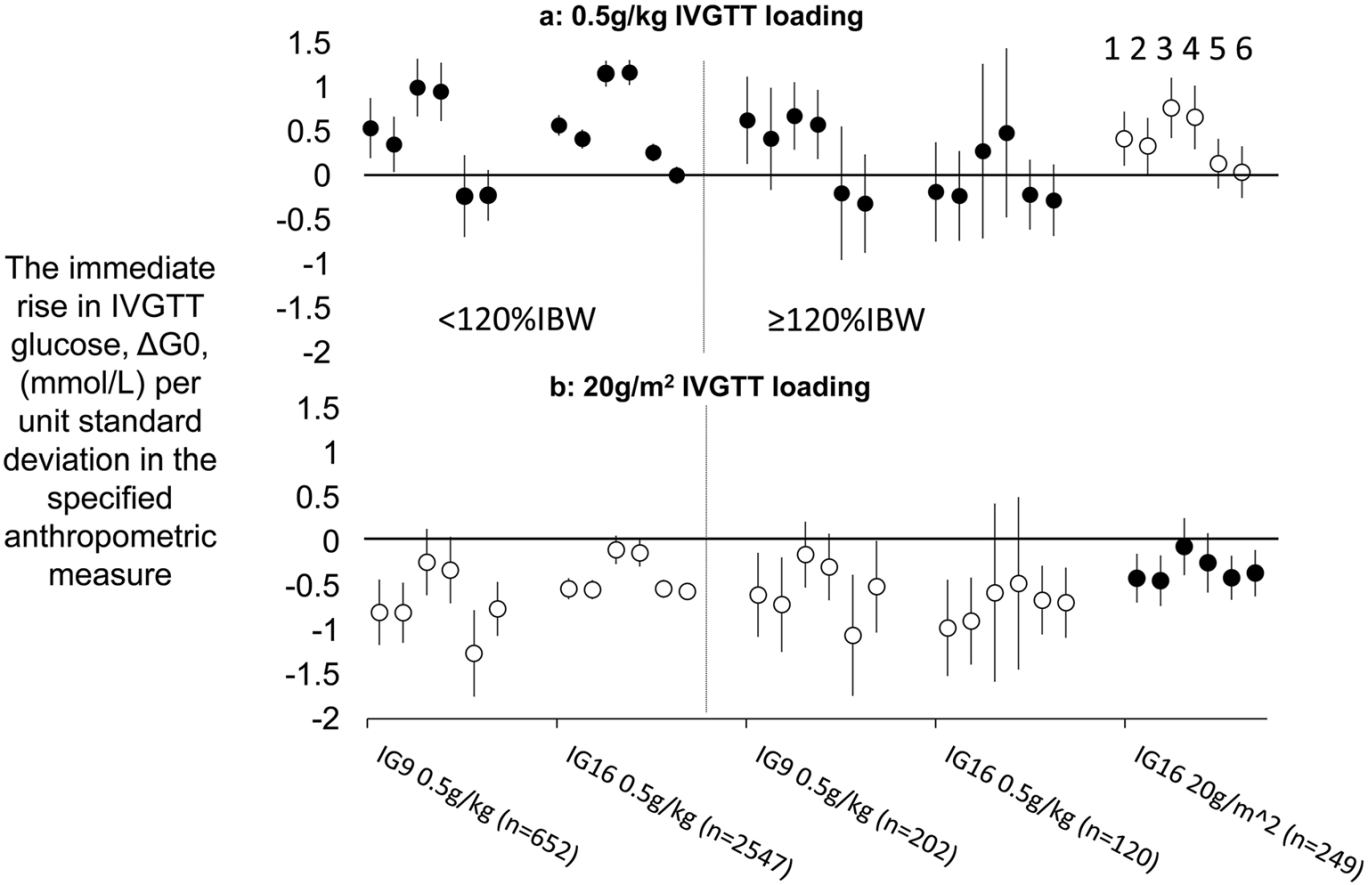
Relationships between the immediate rise in IVGTT glucose concentrations, ΔG0, and six different anthropometric measures, according to IVGTT loadings of 0.5 g/kg or 20 g/m^2^. Markers depict the magnitude of regression coeficients (95%CI) for variation with anthropometric measures in ΔG0, with ΔG0 either observed (filled circles) or calculated (open circles). Calculated values of ΔG0 were derived for each IVGTT by dividing the IVGTT load that would have been given had 0.5 g/kg or 20 g/m^2^ been used by the measured glucose distribution volume. Five groupings were distinguished according to IVGTT source dataset (IG9 or IG16), adiposity (< 120%IBW or ≥ 120%IBW) and actual IVGTT loading received (0.5 g/kg or 20 g/m^2^) and, within each grouping, 6 coefficients are shown depending on which anthropometric measure was entered in the regression. From left to right in each grouping, anthropometric measurements were: (1) weight; (2) body surface area; (3) percent ideal body weight; (4) body mass index; (5) fat free mass; (6) height. Anthropometric variables were standardised to enable comparisons of strengths of association between different anthropometric variables.

Differences in coefficient magnitudes were generally consistent across the 5 groupings although statistical significances were affected to some extent by numbers of observations. With 0.5g/kg loading (Fig. 3a), in those < 120%IBW in both IG9 and IG16 sets (groupings a and b as listed above), strong positive trends with ΔG0 were apparent with the measures of adiposity %IBW and BMI. There were lesser but still highly significant positive trends with ΔG0 for weight and BSA but for FFM and height trends were minimal. With 0.5g/kg loading in those ≥ 120%IBW, in both the IG9 set with observed ΔG0 and the IG16 set with calculated ΔG0 (groupings c and e), positive trends of ΔG0 with weight, BSA, %IBW and BMI were again seen, but the trends appeared weaker for the adiposity measures than in the non-obese. However, in grouping d (IG16 set, ≥ 120%IBW, receiving 0.5 g/kg), there were no associations of ΔG0 with anthropometric measures. This group appeared highly selected since, compared with grouping e (IG16 set, ≥ 120%IBW, receiving 20 g/m^2^): participants were more likely to be women (74 vs 23%), had lower mean %IBW (125 vs. 133%IBW), and were more likely to be participating in HRT studies (46% vs 8%). The group was, in any case, the smallest of the five groupings with only 121 observations. In each of the three groupings for those ≥ 120%IBW with 0.5g/kg loading, there was trend-free variation of ΔG0 with both FFM and height. In contrast to 0.5g/kg loading, trends in ΔG0 with 20g/m^2^ loading were consistent across each of the 5 groupings, being significantly negative for associations of ΔG0 with weight, BSA, FFM and height (Fig. 3b) and non-significant for the adiposity measures %IBW and BMI.

Determining factors in these trends will be the relationship between glucose distribution volume, gVOL, and the anthropometric measure of body size used to calculate the IVGTT load, and the relationship between gVOL and the anthropometric measure in relation to which variation in ΔG0 is evaluated. With weight-based IVGTT loading, for the observed positive associations of ΔG0 with %IBW to be the case, weight must be increasing with %IBW disproportionately more rapidly than glucose distribution volume, gVOL. On the other hand, with BSA-based glucose loading BSA must be changing more proportionately. These inferences were confirmed in a further regression analysis by the coefficients for the number of standard deviation increases with %IBW in weight, BSA and gVOL: 0.044, 0.035 and 0.030 SD/unit %IBW, respectively (with associations and differences between coefficients all *p <* 0.001). Weight, therefore increased 68% more rapidly than gVOL per unit increase in %IBW, whereas the equivalent increase in BSA was only 16%.

The weight- and BSA-based IVGTT load calculations designed to generate a trend-free ΔG0 are evidently not generalisable across a range of anthropometric variables. This recommends a more fundamental approach to IVGTT glucose loading that involves consideration of the relationship between gVOL and the readily available anthropometric variables that could be used to calculate IVGTT loads that will determine whether a trend-free ΔG0 may be expected. As described in ‘*SUPPLEMENTARY SECTION—investigations into alternative IVGTT loading formulae capable of generating a trend-free ΔG0*’, we investigated relationships between gVOL and each of the 6 anthropometric variables according to 3 different functions and established that a linear relationship with a positive intercept could provide the basis for IVGTT load calculations for each anthropometric measure that would generate a trend-free ΔG0. We further evaluated the generalisability of freedom from trend in the values of ΔG0 generated by each IVGTT loading formula to: (1) associations of ΔG0_trend-free_ with anthropometric measures other than the measure used to calculate IVGTT load_trend-free_; (2) to a dataset other than the dataset used to generate the formulae for IVGTT load_trend-free_; and (3) across sex, adiposity and presence of diagnoses likely to affect carbohydrate metabolism and use of medications likely to affect carbohydrate metabolism. In brief, across alternative anthropometric measures, generalisability was highly variable but the BSA-generated IVGTT load specified by the formula [(BSA × 23.9)–9.60] was best able to generate values of ΔG0 with least trend over the full range of anthropometric measures and this was also the case for the IG9 dataset. We calculated that, with this loading formula, in the IG16 dataset, the trend in ΔG0 with increasing %IBW was reduced to 15% of the trend apparent with 0.5g/kg loading and the equivalent figure for the IG9 set was 16%. However, in the IG16 dataset, differences in trends in ΔG0 according to individual characteristics were apparent, with significantly lower strengths of relationship between ΔG0_trend-free_ and %IBW in men and in those with diagnoses likely to affect carbohydrate metabolism.

### Impact of variation in the immediate rise in IVGTT glucose concentrations, ΔG0, on the acute insulin response to glucose, AIRg

**Figure 4:**
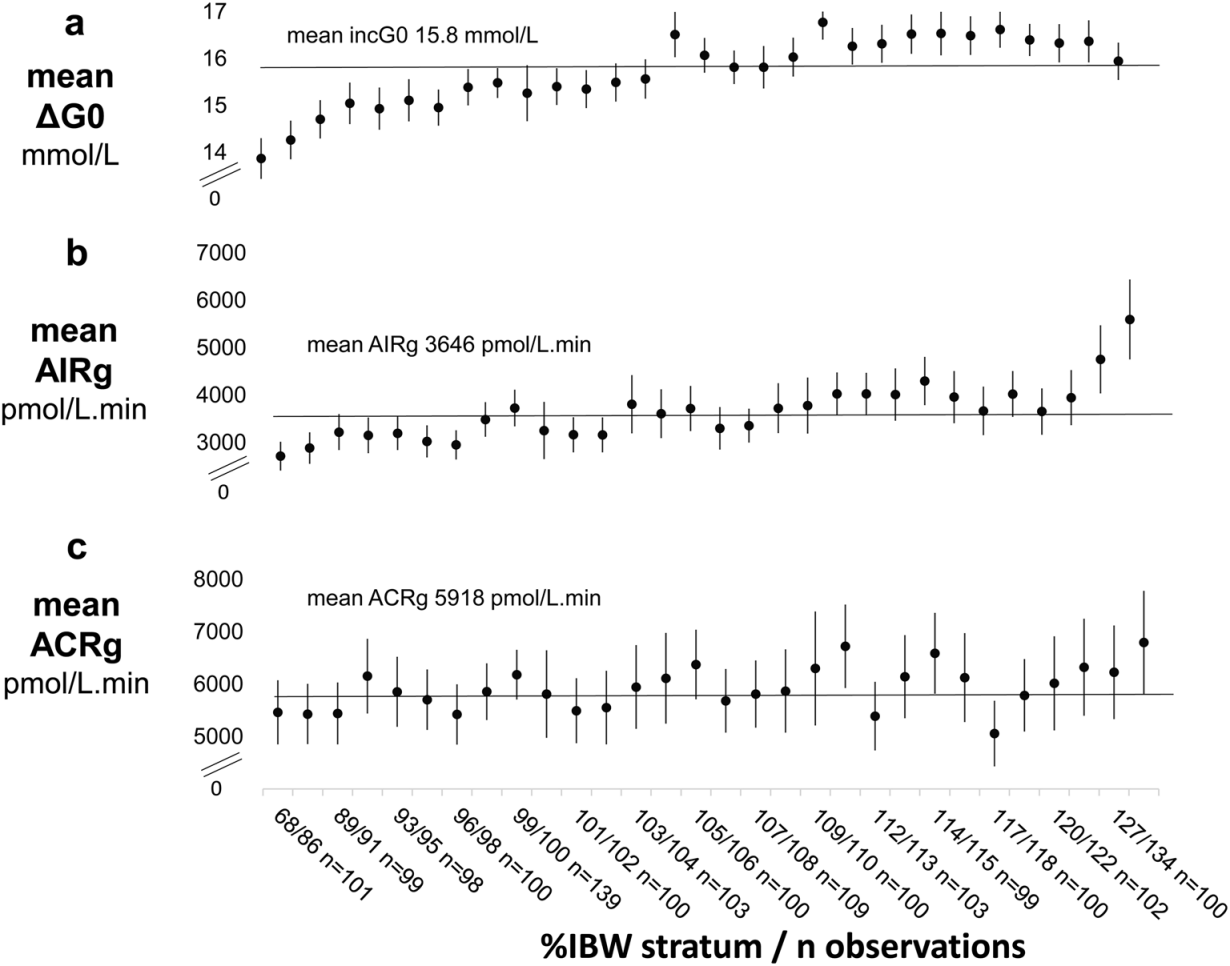
Relationships of the IVGTT immediate rise in glucose concentrations, ΔG0, and the IVGTT acute insulin and c-peptide responses to glucose (AIRg and ACRg) with percentage of ideal body weight (%IBW) as a measure of adiposity. Markers depict the mean (95%CI) for each IVGTT variable in 30 percentile strata of %IBW. The x-axis shows stratum ranges and numbers in each stratum. Percentile-based strata were adopted to optimise similarity in numbers of observations in each stratum Overall, there were 2,951 observations for ΔG0 (Panel a), and for AIRg (Panel b) and 2,566 observations for ACRg (Panel c). NOTE—y axes have been truncated to visualise overall trends.

To assess the extent to which observed trends in ΔG0 with adiposity affected AIRg, variation in mean ΔG0 and mean AIRg were compared in increasing strata of %IBW. The shallow, steady rise in ΔG0 with %IBW that continued to about 120%IBW (Fig. 4a) was closely paralleled by a steady rise in mean AIRg (Fig. 4b). However, above 120%IBW in contrast to the slight fall apparent in ΔG0, there was a marked positive trend in AIRg. Trends in the acute c-peptide response, ACRg, with %IBW were less marked (Fig. 4c) and, although confidence intervals were wide, standardised ACRg showed a significantly weaker relationship with %IBW than did standardised AIRg. Coefficients (95%CI) were 5.7 (2.4, 9.0)*10^–3^ SD per 1% increase in %IBW (*p* = 0.003) for standardised ACRg and 17.7 (14.4, 20.9)*10^–3^ SD per 1% increase in %IBW for standardised AIRg (*p <* 0.001).

Two components of variation in AIRg appeared to be operating: (1) the shallow, steady rise in AIRg with increasing adiposity, which tracked the shallow, steady rise in ΔG0 and (2) the sharp rise in AIRg with increasing overweight and obesity, which had no parallel in variation in ΔG0 nor in ACRg. Nevertheless, that ΔG0 could affect AIRg over its full range was confirmed by the marked, continuous rise in AIRg with ΔG0 (Supplementary Fig. 1)*.* That insulin resistance increased across the full range of adiposity was confirmed by the marked, continuous fall in insulin sensitivity, Si, with increasing %IBW (Supplementary Fig. 2) and that AIRg increased across the full range of increasing insulin resistance was confirmed by the marked, continuous increase in AIRg with decreasing Si (Supplementary Fig. 3)*.*

Regression analysis showed the expected univariable associations of AIRg with IVGTT load (positive), gVOL_measured_ (negative), ΔG0 and %IBW (positive) and Si (negative) (Table 2). In multivariable analysis, ΔG0, %IBW and Si emerged as the independent predictors of AIRg. As might be expected from the formula ΔG0 = IVGTT load/gVOL_measured_ and ΔG0 being the more direct stimulus to insulin secretion, the associations of both gVOL_measured_ and IG load with AIRg were entirely accounted for by ΔG0.

**Table 2:** Associations of the acute insulin response to glucose (AIRg) with potential predictors. Regression coefficients (95%CI)^significance^ are shown for IVGTT load, glucose space (gVOL), the immediate rise in IVGTT glucose concentrations (ΔG0), percentage of ideal body weight (%IBW) and insulin sensitivity, Si, as predictors of the IVGTT acute insulin response to glucose in univariable and multivariable mixed model regression analysis of 2951 IVGTTs for 1,916 participants.

Predictors of AIRg	coefficient (95%CI)^significance^
Univariable	
IVGTT load (g)	82.5 (63.6,101.3)^<0.001^
gVOL (L)	− 126.7 (− 158.7, − 94.7)^<0.001^
ΔG0 (mmol/L)	245.1 (215.8, 274.4)^<0.001^
%IBW	45.7 (38.3, 53.1)^<0.001^
Si (\min\pmol/L)	− 2148.7 (− 2399.0, − 1898.4)^<0.001^
Multivariable	
IVGTT load (g)	− 7.9 (− 46.2,30.4)^0.6^
gVOL (L)	− 13.3 (− 97.2, 70.6)^0.7^
ΔG0 (mmol/L)	239.2 (171.2, 307.1)^<0.001^
%IBW	24.5 (14.8, 34.1)^<0.001^
Si (\min\pmol/L)	− 2081.5 (− 2333.6, − 1829.4)^<0.001^

### Magnitude of the effect on AIRg of variation in the initial IVGTT glucose stimulus, ΔG0

The shallow but continuous rise in ΔG0 with the 0.5 g/kg, weight-determined IVGTT load potentially introduces a methodology-generated confounding factor into measurement of AIRg in that heavier people will receive a proportionately greater initial stimulus to insulin secretion than lighter people. We investigated the magnitude of this potential confounding firstly in non-obese individuals (as conventionally distinguished by BMI < 30 kg/m^2^) by establishing that for every unit increase in BMI, ΔG0 increased by 0.274 mmol/L. A unit increase in ΔG0 was associated with an increase in AIRg of 382 pmol/L min (with variation in insulin sensitivity, Si, also included as a predictor of AIRg to take into account possible adaptation of AIRg to variation in insulin sensitivity). Therefore, for every unit increase in BMI, AIRg would be expected to increase by 105 pmol/L min (0.274 × 382 pmol/L min) due solely to the increasing IVGTT load. The observed increase in AIRg with each unit increase in BMI was 159 pmol/L min/kg/m^2^. Therefore, 47% of the increase in AIRg with BMI in the non-obese was due to increasing IVGTT load. Mean AIRg in the non-obese was 3530 pmol/L min, meaning that AIRg increased by 4.5% of the mean AIRg with each unit increase in BMI and almost a half of this increase was accounted for by the increase in IVGTT load with weight. Among the obese, however (BMI ≥ 30 kg/m^2^), there was no significant increase in ΔG0 with increasing BMI but AIRg increased by 409 pmol/L min per unit increase in BMI (*p <* 0.001), i.e., by 11.6% of the non-obese mean level, entirely independently of IVGTT loading.

Although IVGTT load had an appreciable influence on AIRg in the non-obese, the widespread practice of determining IVGTT load according to weight should at least weaken the influence of variation in gVOL on ΔG0 (and, consequently, AIRg) even if not eliminating it. Accordingly, regression analysis of gVOL alone as a predictor of AIRg should return a weaker, less negative coefficient for gVOL than with gVOL plus IVGTT load both included as predictors of AIRg (which would eliminate any correction for variation in gVOL achieved by IVGTT load). Among all 2951 IVGTTs, this was apparent in the significantly more negative (*p <* 0.001) coefficient for gVOL with both gVOL and IVGTT load as predictors of AIRg (β -322 (95%CI − 365, − 279) pmol/L min, *p <* 0.001) than with gVOL alone as a predictor (β − 130 (95%CI − 166, − 94) pmol/L min, *p <* 0.001).

## Discussion

Across a broad range of weight and adiposity, we found that, among non-obese participants receiving a weight-determined glucose load, the immediate rise in IVGTT glucose concentrations following injection increased with anthropometric measures related to body size, in particular adiposity. Therefore, rather than providing for a comparable glucose stimulus to acute insulin release between participants, the stimulus generated by the weight-determined glucose load increased with increasing adiposity.

To provide for an invariant glucose stimulus to AIRg, the formula for the weight-determined IVGTT load assumes a simple proportionality between weight and glucose space. However, the progressive increase in the glucose stimulus indicates this assumption does not hold and this was confirmed by the 45% faster rate of increase in weight with increasing adiposity relative to the rate of increase in measured glucose volume. Consequently, individuals of higher weight would have received an IVGTT glucose load that was disproportionately high in those of greater adiposity. That this could then exaggerate associations between AIRg and physiological correlates of weight such as adiposity was indicated by the finding that 47% of the increase in AIRg with BMI among the non-obese could be accounted for by the rise in ΔG0 following glucose injection. There was, therefore, a striking consistency between the degree of disproportionality between the increase in weight with adiposity relative to the increase in glucose space with adiposity and the degree of exaggeration in the strength of association between AIRg and adiposity that resulted from the weight-based proportionality function used to calculate the IVGTT load. In the obese, there was also an upward trend in ΔG0 with adiposity, although the association between ΔG0 and adiposity appeared weaker than in the non-obese.

By contrast, the rate of increase in BSA with increasing adiposity was much closer to the rate of increase in glucose space, being only 16% higher. Consequently, any trend in the immediate rise in ΔG0 with adiposity was markedly reduced and, with the 67% admixture of BSA-based glucose loaded IVGTTs in the obese, this resulted in elimination of detectable trend in ΔG0 with adiposity. In support of our hypothesis that a trend-free ΔG0 would allow for an uncompromised evaluation of beta cell function, this trend-free ΔG0 was, nevertheless, associated with a substantial rate of increase in AIRg in the obese, indicating that, rather than IVGTT overloading, the elevated AIRg in the obese must be due to adaptation to the insulin resistance of obesity, either by increased sensitivity of the beta cells to glucose or by reduced insulin elimination, as previously reported^15,16^. The shallower rate of increase in the acute c-peptide response to glucose relative to the insulin response indicated that both mechanisms could be operating.

It might be questioned why, in the century of use of the IVGTT, considerations as basic as the relationships between IVGTT glucose load, gVOL and ΔG0 and their impact on evaluation of IVGTT insulin response appear to have been so little studied. However, reliable findings may have had to wait until a sufficiently large body of IVGTT data with substantial numbers with glucose concentrations below the cut-off for diabetes could be assembled. A major strength of our analysis was that it incorporated an unprecedently large number of observations that could provide for robust identification of relationships between ΔG0, AIRg and measures of body size and a more secure basis for evaluating and projecting alternative IVGTT loading schedules than a smaller sample would have provided.

In designing these analyses, inclusion was determined based on IVGTT validity in relation to the questions to be investigated. Accordingly, IVGTTs associated with negative values of AIRg and fasting plasma glucose concentrations in the diabetic range were excluded. Otherwise, exclusions were based on the methodological considerations of missing data, statistical outliers and minimal model analysis failing to meet established criteria. No constraints were placed on the characteristics of the individuals represented. As well as healthy volunteers, participants in pharmaceutical trials or with a range of clinical conditions were included and this heterogeneity of characteristics is clear in the marked differences in individual characteristics between IG9 and IG16 datasets. Nevertheless, despite these differences, and the different IVGTT sampling schedules, there were striking similarities between IG16 and IG9 datasets in the patterns of relationships of the IVGTT-derived measures, gVOL and ΔG0, with glucose load and anthropometric variables (e.g. Fig. 2c,d and Supplementary Section Fig. 5). These observations support the validity and generalisability of our findings. It should be noted that the IVGTT measures we explored derived from minimal model-based prediction of the underlying IVGTT glucose decay profile rather than individual time points so, providing samples were taken with sufficient frequency and breadth of timing for a valid modelling analysis, estimates of the basic physiological variables, gVOL and ΔG0 should be relatively unaffected, as was apparent in the relative similarity between IG16 and IG9 sets in median values for gVOL and ΔG0.

The IVGTT loading formulae that could best achieve an invariant ΔG0 were those derived using a model of variation in gVOL with anthropometric measures of body size that assumed a linear relationship but with a positive gVOL intercept. This represents a non-physiological simulation, given that it allows for a positive gVOL at zero body size. As described in the Supplementary section, above the minimum anthropometric measures of body size recorded, relationships between gVOL and anthropometric measures were essentially linear. Allowing for a positive gVOL intercept then introduced a degree of flexibility in regression prediction over the actual range of anthropometry that constraining the regression fit to zero gVOL at zero body size excluded. Allowing for a positive intercept could, therefore, offer a simple, practical measure for calculating IVGTT loads that would minimise confounding by a failure to achieve a trend-free initial glucose stimulus to insulin secretion, ΔG0. The generalisability of the formulae to: (a) associations with anthropometric variables other than the variable used to calculate the IVGTT load; (b) a different dataset and (c) groups with different clinical characteristics, nevertheless, appeared highly variable. But, overall, BSA-based calculations showed relatively low trends, potentially within acceptable limits depending on study design. This supports our hypothesis that there would be an optimum anthropometry-based IVGTT load calculation that would achieve a trend-free ΔG0 and accords with the initial observations that BSA might be the optimum variable with which to calculate IVGTT loads.

In clinical research into the metabolic effects of adiposity that has made use of the IVGTT, attention has mostly focused on AIRg, insulin resistance and hyperinsulinaemia in the obese^17–21^. Our findings support the reassuring conclusions that, among the obese, there has been relatively little confounding of associations between AIRg, and adiposity and future studies may benefit from a BSA-based glucose load calculation. Moreover, our analysis of the diminution of the strength of association of AIRg with glucose space resulting from use of weight-based glucose loading also demonstrates that the weight-based load calculation is not without value. Nevertheless, studies of variation in AIRg with any degree of adiposity may be usefully analysed and interpreted with some consideration being given to the possible confounding effects of variable IVGTT glucose loading and variation in ΔG0. G0 is a derived parameter of the minimal model of glucose disappearance and, in relation to the actual glucose load given, can provide a measure of glucose space. These measures can then be considered in any IVGTT analysis so that physiological variations in insulin responses of clinical interest can be better distinguished from variations due to glucose space and the glucose load given.

## Methods

### The Wynn database

The Wynn Database holds information on metabolic measurements recorded between 1965 and 2000, initially at the Department of Metabolic Medicine, St Mary’s Hospital Medical School, London and then at the Cavendish Clinic, London, which subsequently became the Wynn Institute of the U.K. National Heart and Lung Institute, Imperial College London. The full Database comprises 29,245 visit records of metabolic information for 13,848 individuals. Measurements recorded include anthropometric variables and plasma glucose and insulin concentrations during a range of investigative procedures, as well as more specialised measurements in limited numbers. Data were acquired for a variety of participants, including healthy volunteers, endocrine, obesity and lipid clinic patients, coronary heart disease patients and oral contraceptive and HRT users. All methods were carried out in accordance with relevant guidelines and regulations. Ethical approval for continuing data analysis was obtained at national level from the United Kingdom Health Research Authority Central Bristol Research Ethics Committee—Databases (21/SW/0031, 12th April 2021). Because the Wynn Database is a legacy dataset with the last participant contact over two decades ago and with no current participant contact details available the requirement for informed consent for continuing data analysis was reviewed by the United Kingdom Health Research Authority Confidential Advisory Group (21/CAG/0042, 26th April 2021). Continuing data analysis was judged to be in the public interest and the requirement for informed consent was consequently waived by the U.K. Secretary of State for Health under Sect. 251 (4) of the 2006 National Health Service Act.

### Intravenous glucose tolerance test protocol and measurements

All tests were carried out on a metabolic day ward with the participant in a semi-recumbent position. Prior instructions required maintenance of a normal or slightly increased consumption of carbohydrate for 3 days prior to the test, no food to be consumed from 21.00 h the night before the test and arrival at the metabolic day ward between 08.30 and 09.00 h. Anthropometric measurements and clinical details were recorded. Indwelling cannulae were inserted into both cubital veins, one for administration of the glucose load and the other for sampling. Two samples were taken for fasting measurements at 5 min before and immediately prior to commencement of administration of the glucose load. The glucose load was injected over 2–3 min as a 50% glucose solution. Participants < 120%IBW were given an IVGTT load of 0.5 g/kg. For those ≥ 120%IBW, the IVGTT load could be modified to 20 g/m^2^ BSA to reduce the injection burden, although some studies specified that 0.5 g/kg should be used regardless of adiposity. For the present analysis, two IVGTT sampling schedules were distinguished comprising (1) 16 samples: the two fasting samples then sampling at 3, 5, 7, 10, 15, 20, 30, 45, 60, 75, 90, 120, 150 and 180 min following commencement of the glucose injection and (2) 9 samples: two fasting samples then sampling at 10, 20, 30, 40, 60 and 90 min. Samples were placed on ice and plasma was separated within 30 min of the sample being taken. Plasma glucose concentrations were measured on the day of sampling using glucose oxidase-based methods implemented in-house, with ortho-tolidine as colourimetric reagent from 1965 to 1976^22^ and 4-aminophenazone thereafter^23^. Addition of 0.42 mmol/l to all values prior to the change provided for continuity in glucose standardisation. Samples for insulin measurement were frozen at − 20°C and subsequently measured in batches at intervals of 2–4 weeks. To 1992, insulin was measured by an in-house radioimmunoassay using charcoal separation of free and bound fractions and locally-generated anti-insulin antibodies cross-reacting with insulin propeptides. From 1993 onwards, insulin was measured using a double antibody radioimmunoassay, again non-specific with regard to insulin propeptides, and with comparable results (Guildhay Ltd., Guildford, Surrey, UK). Specific insulin (i.e. plasma insulin measured with a monoclonal antibody not cross-reacting with insulin propeptides) was measured from 1994 onwards using an immuno-chemiluminometric method (Molecular Light Technology Ltd., Cardiff, UK). Correlation coefficients between RIA and specific insulin concentrations for each IVGTT sampling point for 113 IVGTTs for which both RIA and specific insulin were measured ranged between 0.95 and 0.99, all highly significant and likely reflecting prevalent normoglycaemia and low, relatively invariant, circulating proportions of insulin propeptides. Regression coefficients from analyses predicting RIA insulin from specific insulin were applied to convert specific insulin concentrations to RIA insulin concentrations for studies in which all insulin measurements were combined. In the present analysis 625 of the 16 sample IVGTTs had specific insulin concentrations measured and converted. C-peptide was measured from 1984 onwards using a double antibody radioimmunoassay (Guildhay Ltd., Guildford, Surrey, UK).Throughout the 35-year period of data recording, consistent attention was paid to quality control and standardisation, with use of commercially available quality control sera, frozen plasma pools at three levels and with previously-measured samples included in each batch. Between-batch assay coefficients of variation for plasma insulin were 6% or less and for C-peptide 8% or less. For glucose, the figure was less than 2%.

### Data analysis

The immediate rise in IVGTT glucose concentrations that provides the stimulus for the phase 1, acute insulin response to glucose, AIRg, was quantified for each IVGTT as the increment above the fasting glucose concentration in G0, ΔG0. G0 is the IVGTT glucose concentration at time zero that would have obtained had glucose administration and distribution been instantaneous. Use of G0 as a measure of the immediate rise in glucose concentrations avoids potential confounding in the initial glucose concentrations by variation in the time taken to inject the glucose load and mixing of glucose concentrations throughout the glucose distribution space. G0 was derived using the Bergman/Cobelli minimal model of glucose disappearance, which describes the profile of IVGTT glucose concentrations from 10 min (to allow for mixing and distribution of the IVGTT glucose load) to the end of the IVGTT in terms of: (1) the model parameter G0; (2) the sensitivity of glucose disappearance to insulin (Si); (3) the sensitivity of glucose disappearance to glucose itself (‘glucose effectiveness’, Sg); and (4) an index of hepatic glucose production^6^. Based on observations reported in previous studies^24,25^, a systematic approach to implementation of the minimal model was adopted for all 16-sample IVGTT glucose and insulin profiles represented in the Wynn Database. A mixing phase of 10 min after glucose injection was allowed in all cases before modelling commenced, after which four different implementations were applied as follows: (1) no constraints and no imputation of measurements beyond 180 min; (2) no constraints, but with imputation of glucose and insulin measurements at 360 min post-injection, with the imputed glucose concentration taken to be the mean of the fasting and 180 min concentrations and the imputed insulin concentration the lowest insulin encountered during the IVGTT; (3) no imputation, but with the fitted glucose concentration constrained to pass through the observed concentration 10 min post-injection; (4) both imputation and constraint applied. After exclusion of modelling analyses returning parameter coefficients of variation (PCV) for Si or Sg of ≥ 100%, parameters from the implementation returning the lowest PCV for Si were selected for inclusion in the Database. Implementation of the minimal model in the 9- sample IVGTTs was according to the method of Cutfield et al. for 90 min duration IVGTTs^26^.

AIRg was calculated as the increment above the fasting level in the trapezium rule-calculated area under the IVGTT insulin concentration profile from 0 to 10 min after the start of the IVGTT. It might be argued that, like the initial glucose concentrations, AIRg is subject to confounding by variation in delivery and mixing of the IVGTT load. It is, therefore worth noting that the Wynn Database holds records for parameters for 504 IVGTTs generated by a minimal model of post-hepatic insulin delivery, which includes I0, the plasma insulin concentration that would have been generated if the first phase bolus output and distribution of insulin by the pancreas had been instantaneous^27^. The correlation between I0 and AIRg was 0.83 (*p <* 0.001), supporting use of AIRg as an effective surrogate for the first phase bolus output of insulin in response to the immediate glucose stimulus, ΔG0. The acute C-peptide response to glucose, ACRg, was also calculated as the increment above the fasting level in the area under the IVGTT C-peptide concentration profile from 0 to 10 min after the start of the IVGTT to provide an index of insulin secretion rather than the index of post-hepatic insulin delivery provided by AIRg.

Anthropometric variables explored in the analysis included: weight; BSA calculated using the Mosteller formula: *BSA* = *square root ((height x weight)/3600)* with height in cm and weight in kg; %IBW determined using ideal body weights derived from Metropolitan Life tables^13^; and body mass index (BMI) was calculated as *BMI* = *weight / height*^*2*^. Fat mass (FM) was calculated using the formula of Gallagher and colleagues^28^: [*FM* = weight*(64.5−(848*(1/BMI)) + (0.079*age)−(16.4*sex) + (0.05*sex*age) + (39.0*sex*(1/BMI)))/100] (with male = 1 and female = 0). Fat-free mass (FFM) was then calculated as the difference between FM and weight^29^. The Wynn Database includes, for 1,368 individuals, dual energy X-ray absorptiometry (DXA) records of total body fat and fat-free masses with which the formulae for FM and FFM could be validated in the context of the present analysis. The correlation between DXA and calculated FM was 0.87 (*p <* 0.001) and between DXA lean mass and calculated FFM 0.95 (*p <* 0.001).

Calculations for IVGTT load, ΔG0 and gVOL, were derived according to arrangements of the formula: gVOL = IVGTT load/ΔG0. The large numbers of observations available for analysis enabled visualisation of the fine structure of associations between these variables and variables upon which they might depend by plotting means and 95%CIs for the dependent variable in strata of the independent variable with strata widths either regular or selected to minimise differences in numbers of observations between strata. Associations between variables were investigated in each full dataset using mixed model regression. Linear and non-linear regression were used with first records for an IVGTT for each participant to derive coefficients for estimation of gVOL from anthropometric variables.

The Wynn Database includes 5763 IVGTT glucose records. For the present analysis, IVGTTs were excluded if they lacked a record of insulin concentrations or they were associated with: (a) negative values for AIRg (indicating measurement error, extreme disruption of beta cell function or problems administering the IVGTT load), (b) exclusion of IVGTT load, ΔG0 or gVOL values outside 3 standard deviations from the mean; (c) minimal model identifications failing to meet validity criteria (parameter coefficients of variation for Si or Sg ≥ 100%), and (d) fasting plasma glucose concentrations ≥ 7.0 mmol/L (indicating severe beta cell deficiency).

### Supplementary Information

Supplementary Information.

## Data Availability

Proposals for data sharing and access to selected data exports in the context of collaborative analyses should be addressed to NO. Because of restrictions based on personal privacy regulations, data cannot be made freely available in a public repository.
