# Supplementary material for: The importance of intravenous glucose tolerance test glucose stimulus for the evaluation of insulin secretion
Source: Sci Rep. 2024 Mar 28;14:7451. doi: 10.1038/s41598-024-54584-x (PMC10978989; doi:10.1038/s41598-024-54584-x)

**The importance of intravenous glucose tolerance test glucose stimulus**

**for the evaluation of insulin secretion**

Ian F Godsland, Desmond G Johnston, KGMM Alberti, Nick Oliver

**SUPPLEMENTARY MATERIAL**

**Supplementary Table 1**

**Relationships between the immediate rise in IVGTT glucose concentrations, ΔG0, and six different anthropometric measures, according to IVGTT loadings of 0.5g/kg or 20g/m^2^. Regression coeficients (95%CI)^significance value^ are shown for variation with anthropometric measures in ΔG0, with ΔG0 either observed or calculated. Calculated values of ΔG0 were derived for each IVGTT by dividing the IVGTT load that would have been given had 0.5g/kg or 20g/m^2^ been used by the measured glucose distribution volume. Five groupings were distinguished according to IVGTT source dataset (IG9 or IG16), adiposity (<120%IBW or ≥120%IBW) and actual IVGTT loading received (0.5g/kg or 20g/m^2^) and, within each grouping, 6 coefficients are shown depending on which anthropometric measure was entered in the regression. Anthropometric measurements considered were: 1) weight; 2) body surface area; 3) percent ideal body weight; 4) body mass index; 5) fat free mass; 6) height. Anthropometric variables were standardised to enable comparisons of strengths of association between different anthropometric variables.**

**Supplementary Table 1**

|  | **A: ΔG0 grouping for 0.5g/kg loading** | | | | |
| --- | --- | --- | --- | --- | --- |
| **predictor of ΔG0** | *IG9 <120%IBW obs*  *n=652, 0.5g/kg* | *IG16 <120%IBW obs*  *n=2547, 0.5g/kg* | *IG9 ≥120%IBW obs*  *n=202, 0.5g/kg* | *IG16 ≥120%IBW obs*  *n=120, 0.5g/kg* | *IG16 ≥120%IBW calc*  *n=249, 20g/m^2^* |
| **weight** | 0.54  (0.20,0.0.88)^0.002^ | 0.57  (0.46,0.69)^<0.001^ | 0.63  (0.14,1.12)^0.01^ | -0.02  (-0.74,0.38)^0.5^ | 0.42  (0.12,0.73)^0.007^ |
| **BSA** | 0.36  (0.05,0.67)^0.02^ | 0.42  (0.31,0.53)^<0.001^ | 0.42  (-0.16,1.00)^0.1^ | -0.23  (-0.73,0.281)^0.3^ | 0.34  (0.02,0.662)^0.03^ |
| **%IBW** | 1.00  (0.67,1.33)^<0.001^ | 1,16  (1.01,1.31)^<0.001^ | 0.68  (0.30,1.06)^<0.001^ | 0.28  (-0.71,1.27)^0.5^ | 0.77  (0.43,1.11)^<0.001^ |
| **BMI** | 0.95  (0.62,1.28)^<0.001^ | 1,17  (1.03,1.31)^<0.001^ | 0.58  (0.19,0.97)^0.003^ | 0.49  (-0.47,1.45)^0.3^ | 0.66  (0.30,1.02)^<0.001^ |
| **FFM** | -0.23  (-0.69,0.24)^0.3^ | 0.27  (0.16,0.37)^<0.001^ | -0.19  (-0.95,0.56)^0.6^ | -0.21  (-0.61,0.18)^0.2^ | 0.14  (-0.14,0.42)^0.3^ |
| **Height** | -0.22  (-0.51,0.07)^0.1^ | 0.01  (-0.09,0.11)^0.8^ | -0.31  (-0.87,0.24)^0.2^ | -0.27  (-0.68,0.13)^0.1^ | 0.04  (-0.25,0.34)^0.7^ |
|  | **B: ΔG0 grouping for 20g/m^2^ loading** | | | | |
|  | *IG9 <120%IBW calc*  *n=652, 0.5g/kg* | *IG16 <120%IBW calc*  *n=2547, 0.5g/kg* | *IG9 ≥120%IBW calc*  *n=202, 0.5g/kg* | *IG16 ≥120%IBW calc*  *n=120, 0.5g/kg* | *IG16 ≥120%IBW obs*  *n=249, 20g/m^2^* |
| **weight** | -0.80  (-1.64,-0.43)^<0.001^ | -0.53  (-0.65,-0.41)^<0.001^ | -0.60  (-1.07,-012)^0..01^ | -0.97  (-1.52,-0.43)^<0.001^ | -0.41  (-0.69,-0.13)^0.004^ |
| **BSA** | -0.80  (-1.14,-0.46)^<0.001^ | -0.54  (-0.64,-0.43)^<0.001^ | -0.71  (-1.24,-0.17)^0.009^ | -0.89  (-1.38,-0.40)^<0.001^ | -0.44  (-0.72,-0.15)^0.003^ |
| **%IBW** | -0.23  (-0.60,0.15)^0.2^ | -0.09  (-0.25,0.07)^0.2^ | -0.14  (-0.51,0.23)^0.4^ | -0.57  (-1.58,0.43)^0.2^ | -0.05  (-0.37,0.27)^0.7^ |
| **BMI** | -0.32  (-0.69,0.06)^0.09^ | -0.12  (-0.28,0.03)^0.1^ | -0.28  (-0.66,0.09)^0.1^ | -0.47  (-1.44,0.51)^0.3^ | -0.24  (-0.57,0.10)^0.1^ |
| **FFM** | -1.26  (-1.75,-0.77)^<0.001^ | 0.53  (-0.63,-0.43)^<0.001^ | -1.06  (-1.74,-0.37)^0.002^ | -0.66  (-1.04,-0.27)^0.001^ | -0.40  (-0.65,-0.16)^0.001^ |
| **Height** | -0.76  (-1.06,-0.45)^<0.001^ | -0.56  (-0.66,-0.46)^<0.001^ | -0.50  (-1.02,0.02)^0.05^ | -0.69  (-1.08,-0.29)^0.001^ | -0.35  (-0.61,-0.09)^0.009^ |

**Supplementary figure 1. Relationship between the IVGTT acute insulin response to glucose (AIRg) and the immediate rise in IVGTT glucose concentrations, ΔG0. Markers depict mean (95%CI) for AIRg in 30 percentile strata of the immediate rise in IVGTT glucose (ΔG0) for 2,951 IVGTTs for participants with ΔG0 in the range 8.2-22.9 mmol/L, with x-axis stratum ranges defined to optimise, as shown, the similaritybetween strata in numbers of observations recorded.**


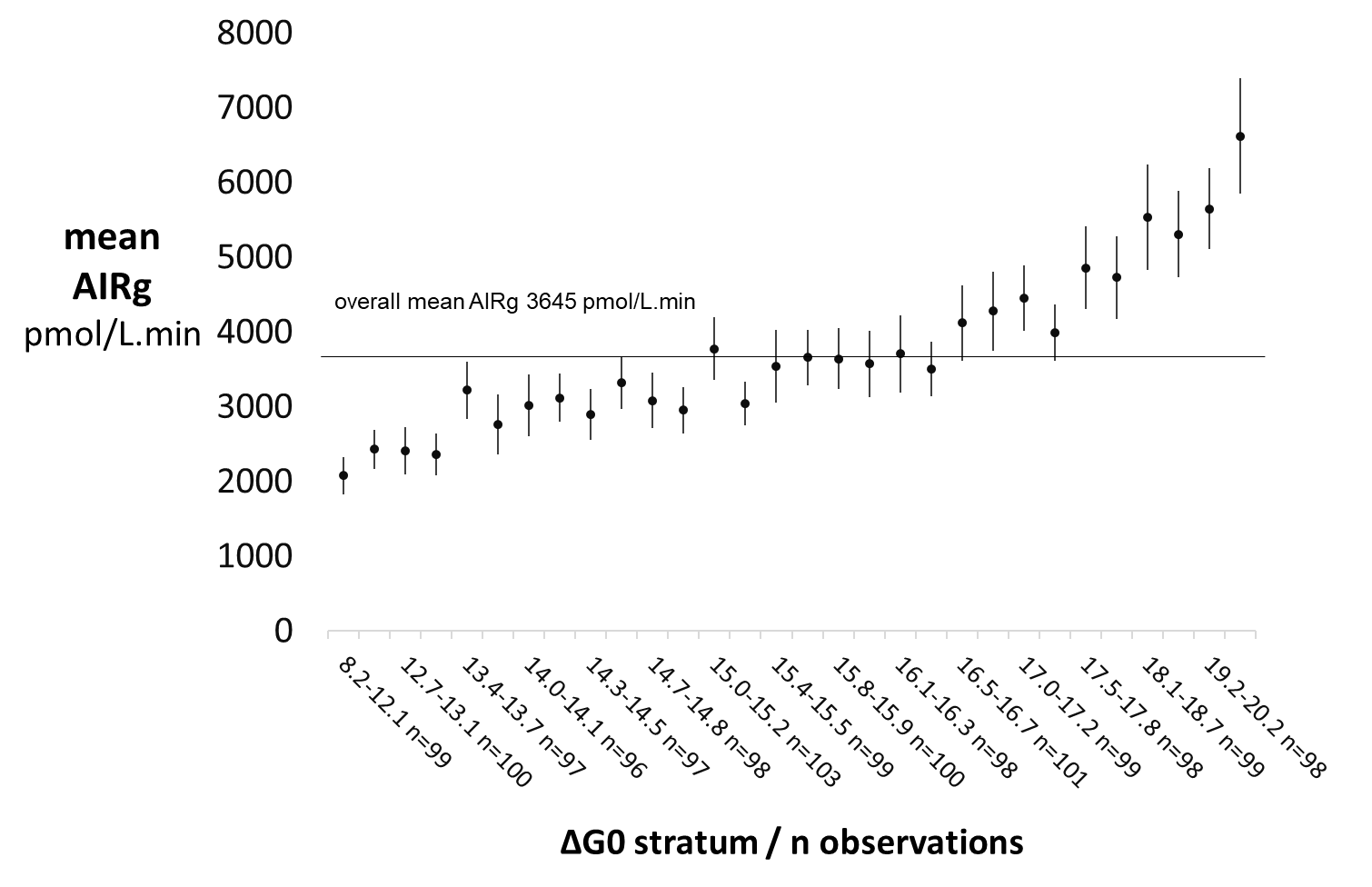


**Supplementary figure 2. Relationship between insulin sensitivity, Si, and percentage of ideal body weight (%IBW) as a measure of adiposity. Markers depict mean (95%CI) for insulin sensitivity (Si) in 30 percentile strata of percentage of ideal body weight (%IBW) for 2,951 IVGTTs for participants with %IBW in the range 68-192%, with stratum ranges defined to optimise similarity in numbers of observations in each stratum**


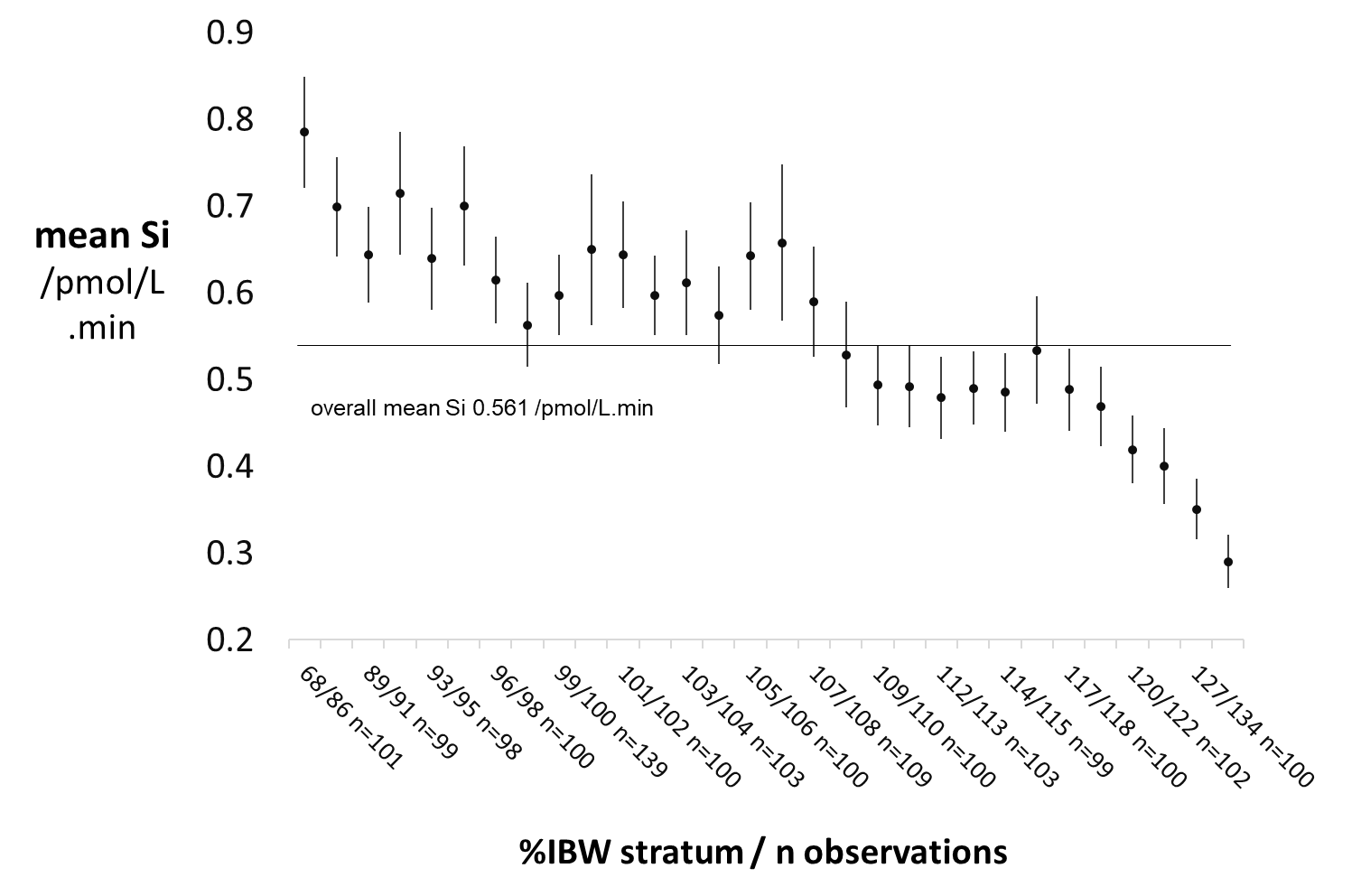


**Supplementary figure 3. Relationship between the acute insulin response to glucose, AIRg, and insulin sensitivity (Si). Markers depict mean (95%CI) for the acute insulin response to glucose (AIRg) in 30 percentile strata of insulin sensitivity (Si), for 2,951 IVGTTs for participants with Si in the range 1.17–0.05 \pmol/L.min (i.e. least to greatest insulin resistance), with stratum ranges defined to optimise similarity in numbers of observations in each stratum**


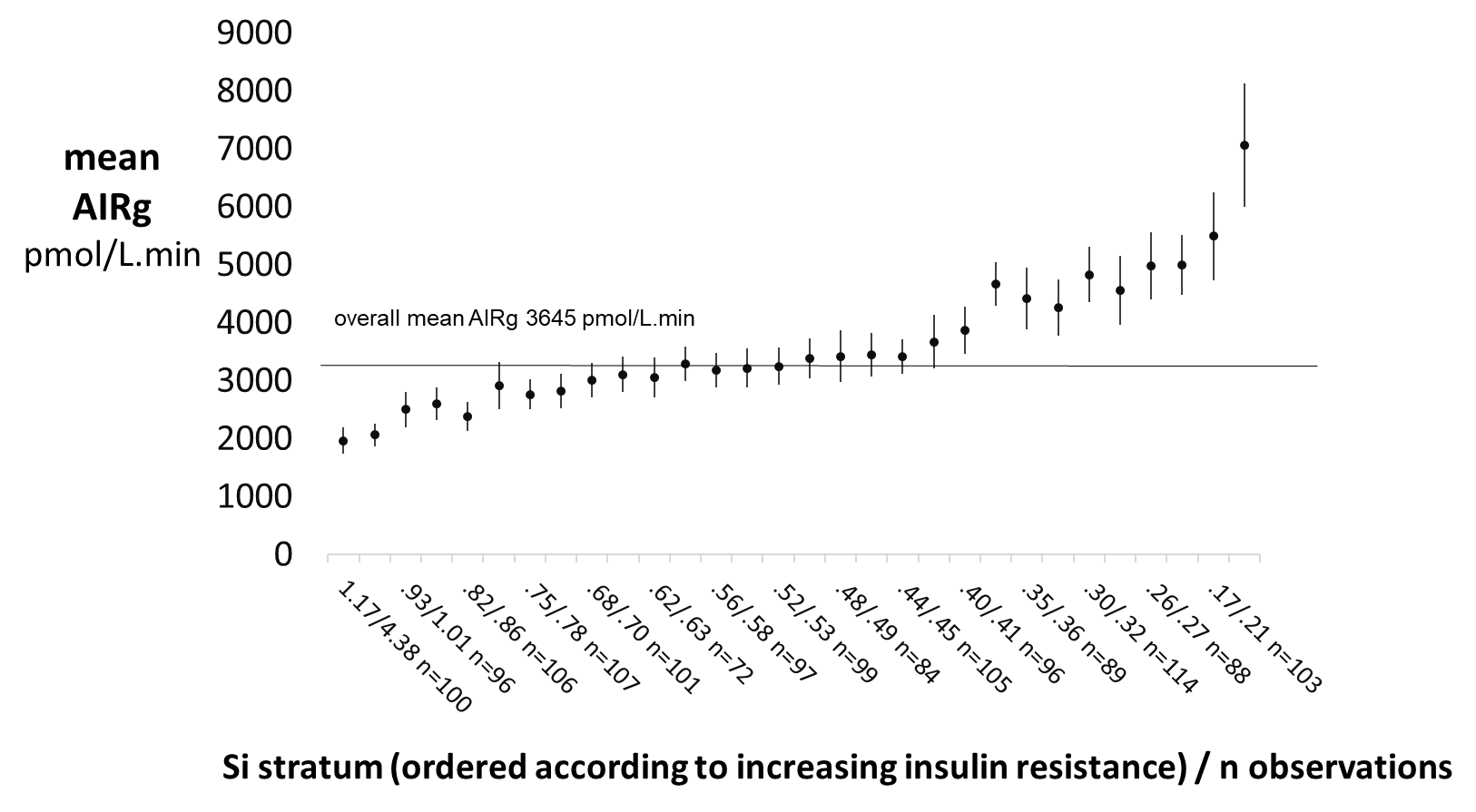


**SUPPLEMENTARY SECTION** **– investigations into alternative IVGTT loading formulae capable of generating a trend-free ΔG0**

***Relationships between glucose distribution volume and readily available anthropometric measures of body size***

If a measure of glucose distribution volume, gVOL, is available, the IVGTT load that will generate a trend-free immediate rise in IVGTT glucose concentrations (ΔG0: the stimulus for the acute insulin response to glucose) can be calculated as IVGTT load_trend-free_ = gVOL x ΔG0_trend-free_, where ‘ΔG0_trend-free_‘ can be any chosen magnitude of rise in ΔG0. However, given that gVOL is generally not available, an IVGTT load calculation based on a surrogate index of gVOL provided by a readily available anthropometric measure of body size may be considered. How successful such an anthropometric measure will be in providing for an effective calculation of IVGTT load_trend-free_ will then depend on identification of a function that can accurately relate gVOL to the anthropometric measure.

Both 0.5g/kg body weight and 20g/m^2^ body surface area (BSA) IVGTT load calculations assume that weight and BSA relate to gVOL according to a linear proportionality function with gVOL equal to zero when weight or BSA is zero. This function is given by: gVOL= β1 x weight or gVOL= β2 x BSA where β1 and β2 are the proportionality constants. For generating trend-free variation in ΔG0, the formula: IVGTT load_trend-free_ = gVOL x ΔG0_itrend-free_ must obtain: Therefore, for weight-based IVGTT loading, IVGTT load_itrend-free_ is presumed to be equal to β1 x weight x ΔG0_itrend-free_. The two terms: β1 and ΔG0_trend-free_ are constants_,_ so their product is itself a constant. Therefore, IVGTT load_trend-free_ is equal to β3 x weight, where β3 =β1 or β2 x ΔG0_itrend-free_. For the 0.5g/kg weight-based glucose load calculation, β3 takes a value of 0.5 and for the 20g/m^2^ BSA-based calculation, a value of 20. But these formulations will only provide for a trend-free ΔG0, if there is, in fact, strict proportionality between gVOL and weight or BSA.

As summarised in Figure 3 of the main text, 0.5 g/kg loading tends to generate positive relationships between ΔG0 and measures of body size and 20 g/m^2^ loading tends to generate negative relationships. As illustrated in Figure 2, for 0.5 g/kg loading, the positive associations appear to be generated by underestimation the IVGTT load needed to generate an invariant ΔG0 at low body size and overestimation at high body size. Accordingly, the linear model of the relationship between gVOL and body size represented by 0.5 g/kg loading underestimates gVOL at low body size and overestimates gVOL at high body size. For 20 g/m^2^ loading and the negative associations it generates, the converse is the case. These considerations suggest that power function relationships between gVOL and anthropometric variables may be better able to generate an invariant ΔG0 than linear relationships. As illustrated in *Supplementary section Figure 1*, it can be seen how, if a power function represents the true relationships of gVOL with weight, the assumption of linear proportionality will lead to underestimation of gVOL at low weight – and, therefore, underestimation of the IVGTT glucose load needed to establish a trend-free ΔG0 – and overestimation of gVOL at high weight – and, therefore, overestimation of the IVGTT glucose load needed to establish a trend-free ΔG0. Under- and over-estimation then combine to generate the observed positive association between ΔG0 and weight. It can also be seen how, for BSA, the opposite biases would be the case.

**Supplementary section Figure 1. Glucose distribution volume, gVOL, predicted by linear (dashes) and power function (lines) relationships with weight and BSA. Calculation coefficients shown were generated by linear or non-linear regression of IVGTT-measured gVOL on weight or BSA. If the continuous lines generated by the power functions represent the true relationships between gVOL and weight or body surface area, it can be seen how the assumption of linear relationships implicit in the IVGTT loadings of 0.5g/kg body weight or 20g/m^2^ body surface area will under- or over-estimate gVOL.**


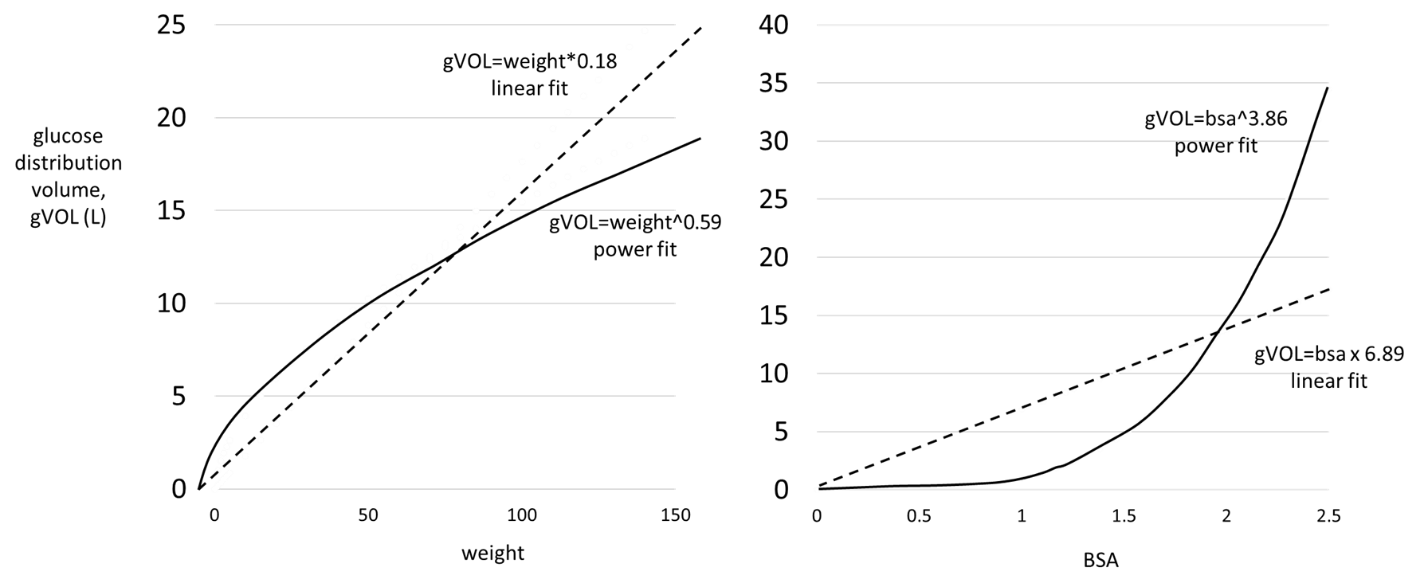


We investigated relationships between measured gVOL and the six anthropometric measures of body size: weight; BSA; percentage of ideal body weight (%IBW); body mass index (BMI); fat-free mass (FFM); and height and their use in alternative calculations of IVGTT load_itrend-free_ based on three simple functions, each relating gVOL to each anthropometric measure:

a) *linear no constant* (gVOL = β x anthropometric variable: physiologically realistic in that when body size is zero, gVOL will be zero).

b) *power function* (gVOL = anthropometric variable^β^: similarly physiologically realistic)

c) *linear with constant* (gVOL = β x anthropometric variable + constant: not strictly physiologically realistic in that it allows for gVOL to differ from zero body size but potentially offering a more flexible approximation to an accurate prediction of gVOL).

Using the principal IG16 set, gVOL was estimated using regression coefficients from each of these three functions to predict measured gVOL from each of the six anthropometric measures. Coefficients were then derived for standardised values for each anthropometric measure as a predictor of the differences between the measured and estimated values of gVOL (i.e. gVOL_estimated_ minus gVOL_measured_) as shown in *Supplementary section Figure 2*.

**Supplementary section Figure 2. Coefficients (95%CI) for variation in the difference between measured glucose distribution volume, gVOL_measured_ and estimated gVOL, gVOL_estimated_ relative to the anthropometric measure used to generate gVOL_estimated_. Estimation was according to 3 different functions with 6 different anthropometric estimators of gVOL_measured_. From left to right for each of the 3 functions anthropometric estimators were: 1) weight; 2) body surface area; 3) percent ideal body weight; 4) body mass index; 5) fat free mass; and 6) height. Measured gVOL derived from 2,951 IVGTTs.**


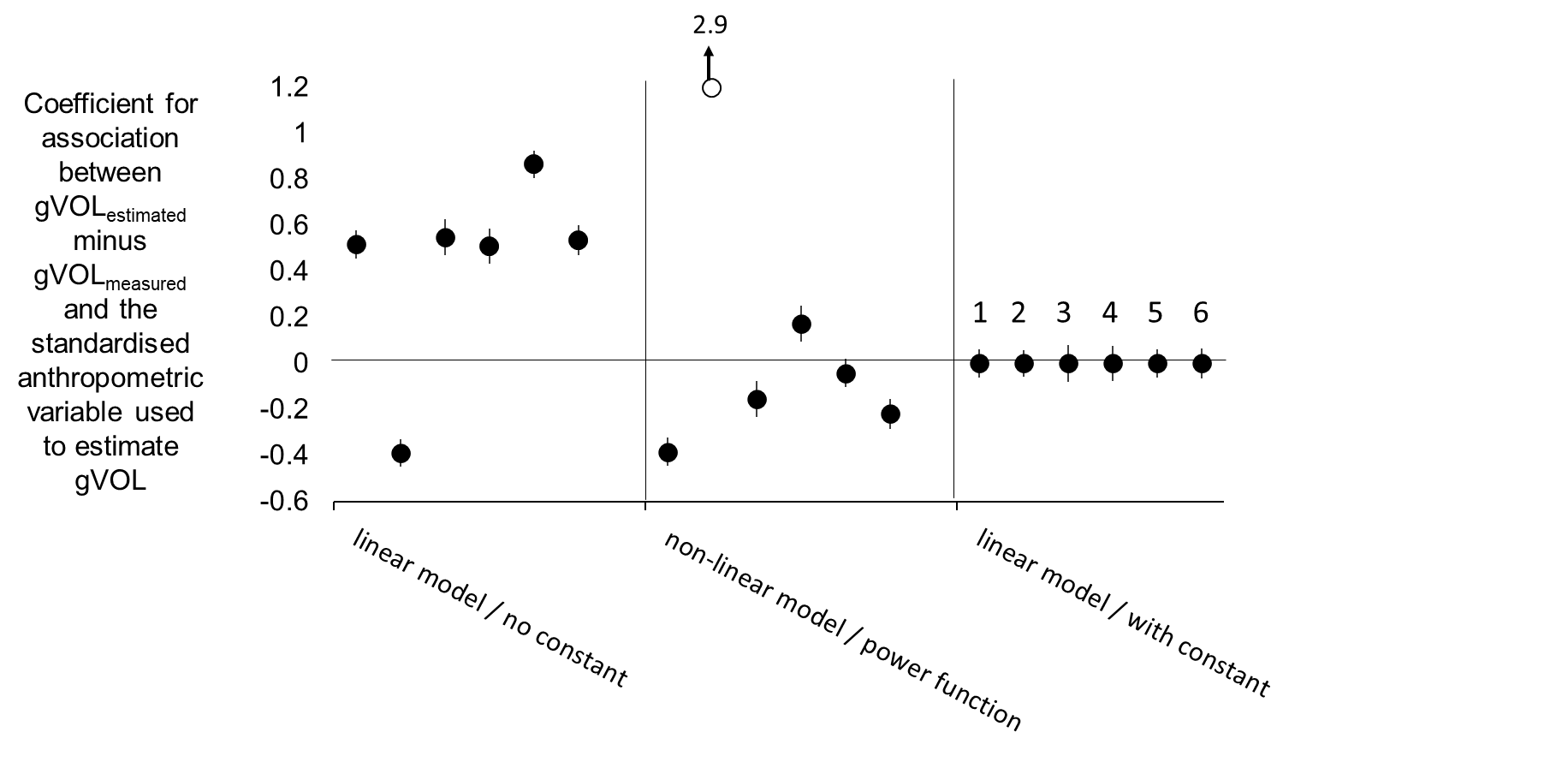


The linear, no-constant function (as implicit in the 0.5g/kg and 20g/m^2^ loadings) returned estimates of gVOL that generated a highly significant, positive trend in the differences between gVOL_estimated_ and gVOL_measured_ with all anthropometric variables except BSA, which returned a highly significant negative trend. With power function-based estimates of gVOL, biases in the differences between gVOL_estimated_ and gVOL_measured_ were markedly reduced in strength compared with those generated by the linear function with no constant but tended to be negative, although BSA was again an exception, returning an acutely positive trend. However, the linear function with a constant generated estimates of gVOL that showed the desired zero trend in the differences with variation in the anthropometric predictor measures, indicating that gVOL_estimated_ was free of bias relative to the variable used in the estimation. Taking weight-estimated gVOL as a typical example, profiles for variation of the differences between gVOL_estimated_ and gVOL_measured_ with weight generated by the 3 functions are illustrated in *Supplementary section Figure 3*.

**Supplementary section Figure 3. Means (95%CI, n=2,951) for the difference between measured glucose distribution volume, gVOL, and gVOL estimated by 3 different functions of weight in 30 percentiles of increasing weight. Weight ranges for every other stratum of weight are shown with numbers of observations in each stratum.**


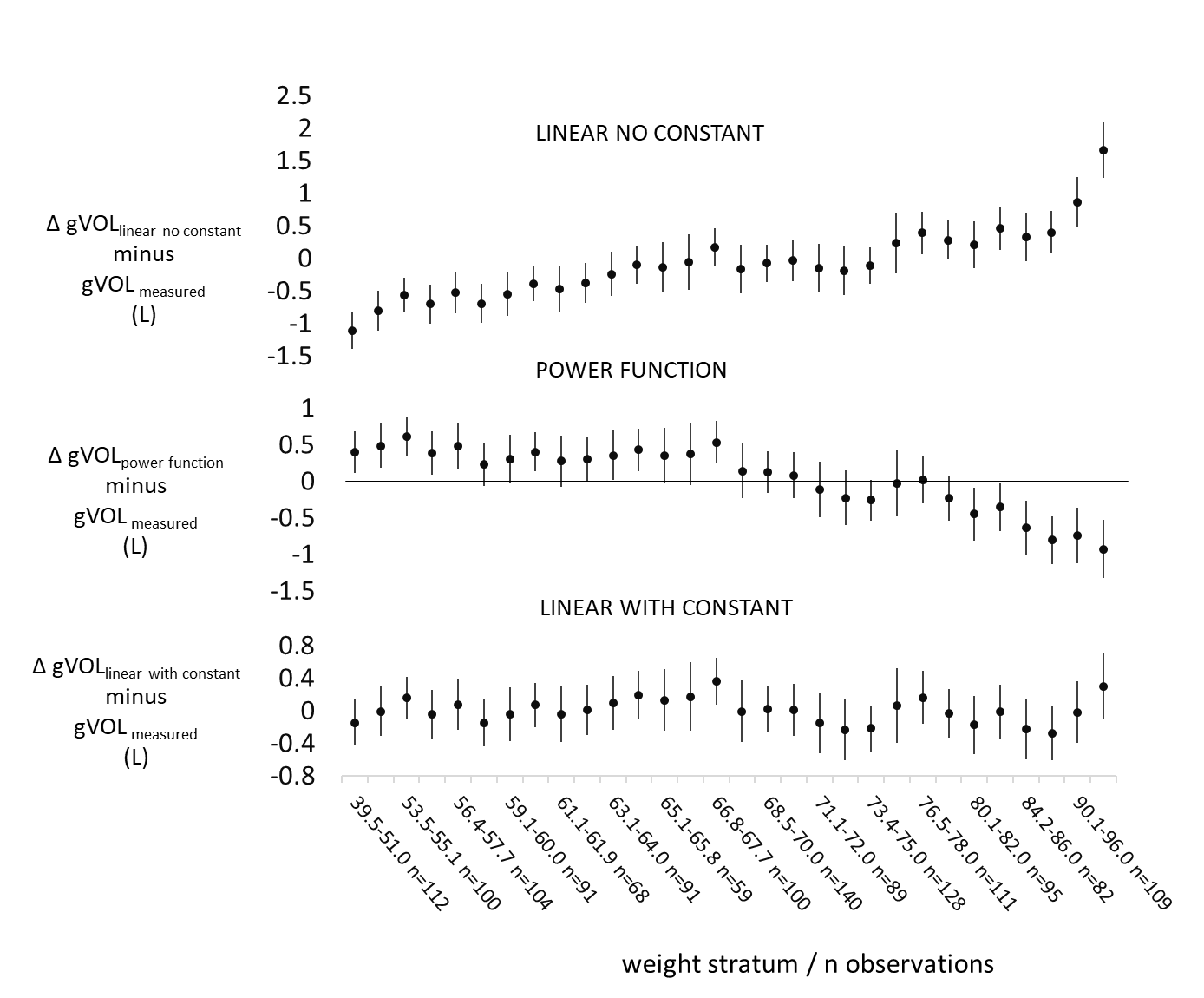


Rather than there being a linear relationship between the glucose distribution volume and weight, the rise in ΔG0 with increasing IVGTT load appeared more consistent with a power function relationship, although increasing bias was apparent at high weight. Rather than a power function, a linear relationship with positive intercept appeared to be best able to sustain a trend-free ΔG0. Above the minimum body weight of 30 kg, it, therefore, appears that the true relationship between gVOL and weight may be virtually linear, as can be seen in *Supplementary section Figure 4.* Possibly, in having to accommodate the initial curvature in the relationship between gVOL and weights between zero and 30 kg, the power function may have to generate excessive curvature in the relationship above 30 kg, leading to overestimation of gVOL at low weight and underestimation at high weight and, consequently, a negative relationship between gVOL_estimated_ minus gVOL_measured_ and weight.

**Supplementary section Figure 4. Glucose distribution volume, gVOL, predicted by 1) linear function with no intercept (dashes); 2) power function (line); and 3) linear function with positive intercept (bold dashes). Calculation coefficients shown were generated by linear or non-linear regression of IVGTT-measured gVOL on weight. The profiles demonstrate how the linear function with positive intercept can correct for observed over- and under-estimation of gVOL by the linear function with no intercept or by the power function in the actual weight-range 39-150kg.**


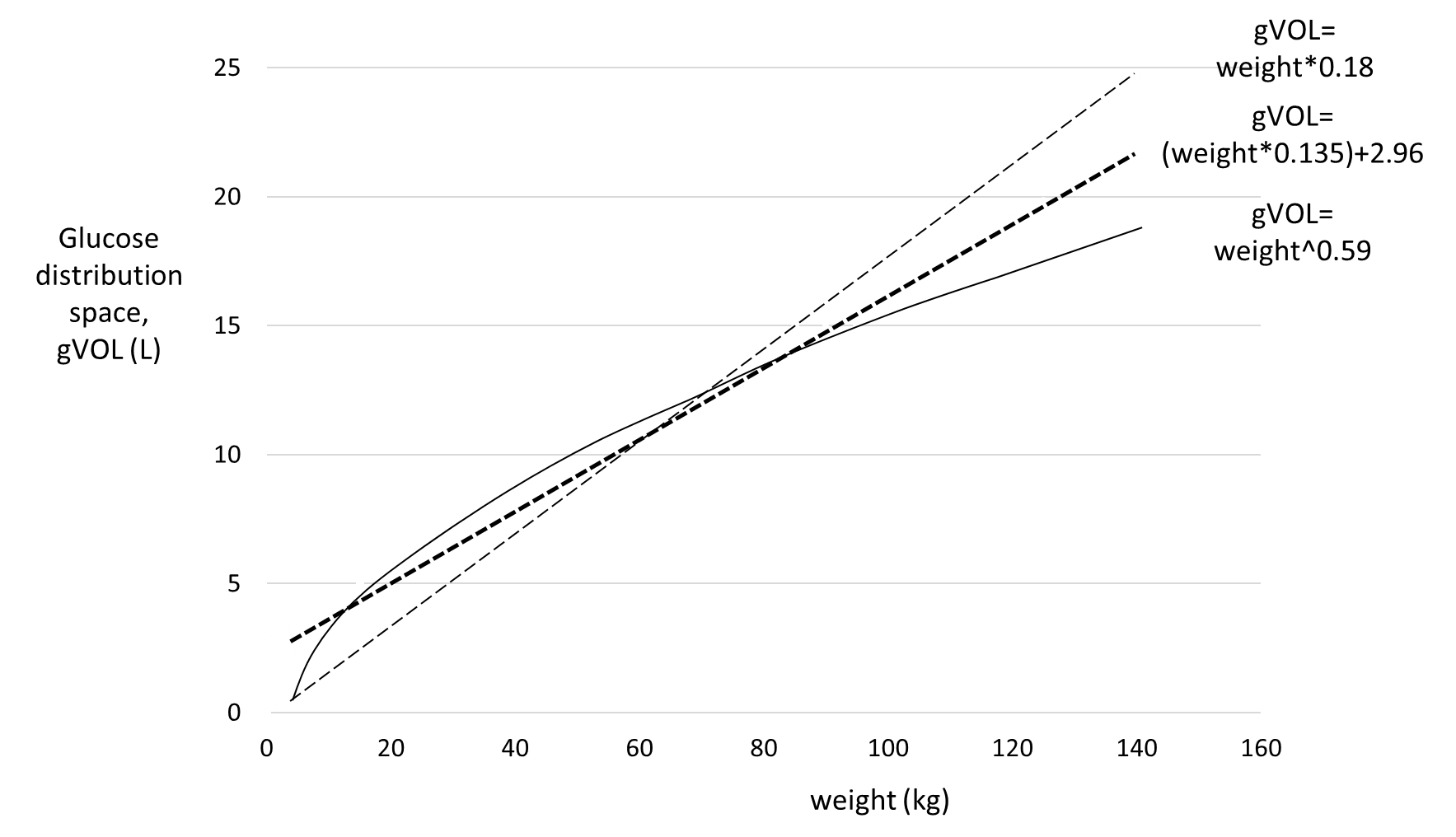


***Calculations for the IVGTT loads needed to*** ***generate a trend-free immediate rise in IVGTT glucose, ΔG0, for a range of anthropometric variables.***

For weight, the linear function-with-intercept estimation of gVOL was given by: gVOL = ((weight*0.135)+2.956). The IVGTT load that will generate a trend-free ΔG0 is given by: IVGTT loadt_rend-free_ = gVOL x ΔG0_trend-free_. With ΔG0_trend-free_ set to 15.0 mmol/L, the IVGTT load required for a trend-free ΔG0 of 15mmol/L was then given by IVGTT load_trend-free-weight_ =((weight x 0.135 )+2.956) x ((15 x180)/1000) g, which reduces to IVGTT load_trend-free-weight_ = (weight x 0.365) + 7.982 g. For the other 5 anthropometric measures the formulae were: IVGTT load_trend-free-BSA_ = (BSA x 23.9) -9.60; IVGTT load_trend-free%IBW_ = (%IBW x 0.198) +12.251; IVGTT load_trend-free-BMI_ = (BMI x 0.917) +10.985; IVGTT load_trend-free-FFM_ = (FFM x 0.436) + 11.605; and IVGTT load_trend-free-height_ = (height x 0.461) + 8.729. For each anthropometric measure, the validity of the derivation and calculation was checked by dividing IVGTT load_trend-free_ by the measured gVOL to generate ΔG0_trend-free_ and then regressing ΔG0_trend-free_ on the anthropometric variable used to calculate IVGTT load_trend-free_ to confirm no significant trend in ΔG0_trend-free_.

***Generalisability of the IVGTT loads needed to generate a trend-free immediate rise in IVGTT glucose, ΔG0***

*1) Generalisability to trends with anthropometric measures of body size other than the measure used to generate ΔG0_trend-free_*

To determine how generalisable each of the 6 anthropometric measure-generated estimates of IVGTT load_trend-free_ were for generating a trend-free ΔG0 relative to anthropometric measures of body size other than the measure used to generate IVGTT load_trend-free_, the ΔG0_trend-free_ generated by each IVGTT load_trend-free_ was regressed on the 5 anthropometric measures other than the measure used to generate IVGTT load_trend-free_. In these analyses, standardised anthropometric measures were used throughout to enable between-measure comparisons of the strengths of association between ΔG0_trend-free_ and the alternative anthropometric measures. Coefficients and confidence intervals are listed in *Supplementary section Table 1, panel A*.

**Supplementary section Table 1. Regression coefficients (95%CI)^significance^ for standardised anthropometric variables as predictors of ΔG0, generated in the IG16set with IVGTT load_trend-free_ calculated from gVOL estimated by each anthropometric variable and a target, trend-free ΔG0 of 15 mmol/L (n=2,951).**

|  | **A: 1G16 set** | | | | | |
| --- | --- | --- | --- | --- | --- | --- |
| ΔG0 varying with... | *weight-derived ΔG0_trend-free_* | *BSA-derived ΔG0_trend-free_* | *%IBW-derived iΔG0_trend-free_* | *BMI-derived*  *ΔG0_trend-free_* | *FFM-derived ΔG0_trend-free_* | *height-derived ΔG0_trend-free_* |
| **weight** | 0.011  (-0.074,0.097)^0.7^ | 0.041  (-0.041,0.123)^0.3^ | -1.110  (-1.211,-1.009)^<0.001^ | -0.965  (-1.066, -0.863)^<0.001^ | -0.224  (-0.310,-0.138)^<0.001^ | -0.738  (-0.827,-0.650)^<0.001^ |
| **BSA** | -0.088  (-0.173,-0.004)^0.04^ | 0.010  (-0.072,0.091)^0.8^ | -1.324  (-1.419,-1.228)^<0.001^ | -1.187  (-1.284,-1.091)^<0.001^ | -0.212  (-0.297, -0.127)^<0.001^ | -0.599  (-0.689,-0.509)^<0.001^ |
| **%IBW** | 0.393  (0.298,0.488)^<0.001^ | 0.174  (0.081,0.266)^<0.001^ | -0.027  (-0.153,0.098)^0.6^ | 0.064  (-0.060, 0.187)^0.3^ | -0.295  (-0.391,-0.198)^<0.001^ | -1.135  (-1.229,-1.041)^<0.001^ |
| **BMI** | 0.372  (0.279,0.465)^<0.001^ | 0.166  (0.075,0.256)^<0.001^ | -0.167  (-0.291,-0.044)^0.009^ | -0.014  (-0.136, 0.107)^0.8^ | -0.253  (-0.347,-0.158)^<0.001^ | -1.135  (-1.227,-1.043)^<0.001^ |
| **FFM** | -0.173  (--0.254,-0.092)^<0.001^ | -0.036  (-0.114,0.043)^0.3^ | -1.404  (-1.493,-1.315)^<0.001^ | -1.266  (-1.357,-1.176)^<0.001^ | -0.034  (-0.117,0.049)^0.4^ | -0.435  (-0.524,-0.347)^<0.001^ |
| **Height** | -0.404  (-0.487,-0.321)^<0.001^ | -0.113  (-0.194,-0.031)^0.007^ | -1.730  (-1.813,-1.647) ^<0.001^ | -1.659  (-1.743,-1.576)^<0.001^ | -0.124  (-0.210,-0.038)^0.005^ | -0.004  (-0.098,0.090)^0.9^ |
|  | **B: IG 9 set** | | | | | |
| **weight** | 0.289  (0.090, 0.488)^0.004^ | 0.133  (-0.056, 0.321)^0.1^ | -0.348  (-0.596, -0.101)^0..008^ | -0.234  (-0.479, 0.011)^0.06^ | -0.770  (-1.005,-0.535)^<0.001^ | -1.289  (-1.488,-1.091)^<0.001^ |
| **BSA** | 0.149  (-0.071, 0.369)^0.1^ | 0.086  (-0.121,0.293)^0.4^ | -0.727  (-0.994,-0.459)^<0.001^ | -0.616  (-0.881,-0.351)^<0.001^ | -0.894  (-1.151,-0.637)^<0.001^ | -1.247  (-1.472,-1.022)^<0.001^ |
| **%IBW** | 0.449  (0.303, 0.595)^<0.001^ | 0.217  (0.077,0.358)^0.002^ | 0.343  (0.158,0.527)^<0.001^ | 0.386  (0.205,0.567)^<0.001^ | -0.438  (-0.621,-0.256)^<0.001^ | -1.034  (-1.179,-0.889)^<0.001^ |
| **BMI** | 0.430  (0.280,0.580)^<0.001^ | 0.185  (0.040, 0.330)^0.01^ | 0.272  (0.082, 0.463)^0.005^ | 0.372  (0.185,0.558)^<0.001^ | -0.458  (-0.642,-0.273)^<0.001^ | -1.123  (-1.269,-0.976)^<0.001^ |
| **FFM** | -0.422  (-0.781,-0.063)^0.02^ | -0.332  (-0.668, 0.004)^0.05^ | -1.770  (-2.188,-1.351)^<0.001^ | -1.656  (-2.077,-1.235)^<0.001^ | -1.009  (-1.353,-0.666)^<0.001^ | -1.348  (-1.724,-0.973)^<0.001^ |
| **Height** | -0.733  (-0.983,-0.483)^<0.001^ | -0.287  (-0.528, -0.046)^0.01^ | -2.030  (-2.300,-1.760)^0.05^ | -2.061  (-2.323,-1.798) ^<0.001^ | -0.645  (-0.946,-0.345)^<0.001^ | 0.238  (-0.050,0.527)^0.1^ |

Weight-based estimation of IVGTT load_trend-free_ generated a trend in ΔG0 close to zero with BSA but otherwise marked trends with weight-based ΔG0 were discernible (*Supplementary section Figure 5, IG16 set panel*). BSA- and FFM-based estimation of IVGTT load_trend-free_ generated trends close to zero with other anthropometric measures. %IBW and BMI-based estimation of IVGTT load_trend-free_ generated interchangeable zero trends but otherwise trends for adiposity-based estimation of IVGTT load_trend-free_ diverged substantially from zero for other anthropometric measures.

**Supplementary section Figure 5. Coefficients for six standardised anthropometric variables as predictors of the immediate increase in IVGTT glucose, ΔG0, derived from estimated glucose distribution volume. From left to right within each estimation grouping: weight, BSA, %IBW, BMI, FFM, height. Filled circles indicate coefficients for anthropometric variables other than the variable with which gVOL was estimated; open circles for the anthropometric variable with which gVOL was estimated.**


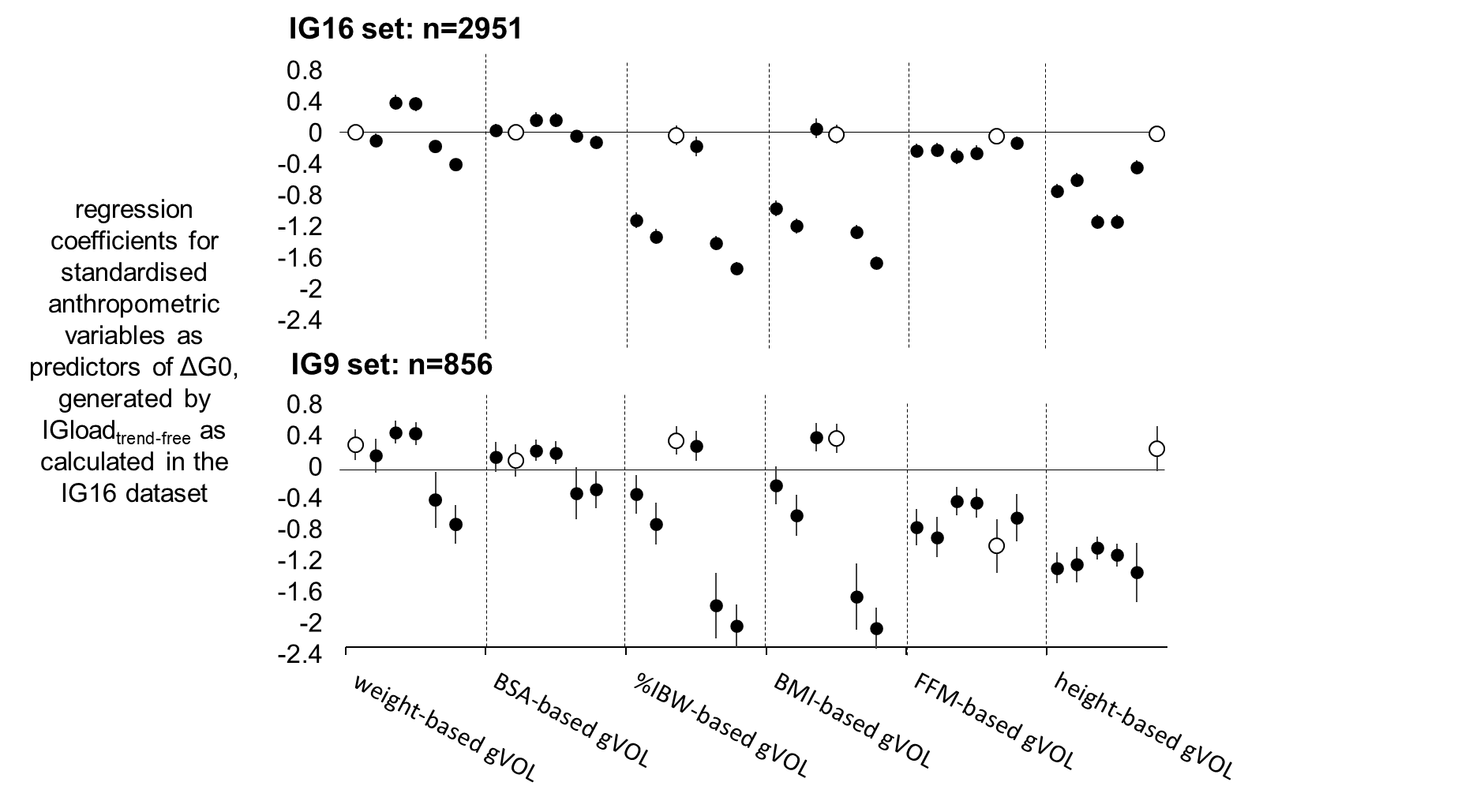


*2) Generalisability in a dataset other than the dataset used to generate ΔG0_trend-free_*

IVGTT load_trend-free_ determined in the IG16 set was further assessed for generalisability by applying it in calculation of ΔG0 in the IG9 set, again using measured gVOL (*Supplementary section Table 1, panel B and Figure 5, IG9 panel*). Trends tended to be relatively close to zero when ΔG0 was regressed on the anthropometric measure of body size used to generate the estimate of IVGTT load_trend-free_ but there was evidence of a residual positive trend. For variation with measures other than the derivation measure, IVGTT load_trend-free-BSA_ showed the fewest overall divergences from zero but, otherwise, there were marked divergences from zero trend.

As an overall index of generalisability, a simple average of the regression coefficients was derived for variation with anthropometry across the six different anthropometric measures of body size in ΔG0 generated by each set of IVGTT load_trend-free_. In the IG16 set, the lowest score was for ΔG0 generated by IVGTT load_trend-free-BSA_, amounting to 0.09 mmol/L per unit change in SD, which compared favourably with an average of 0.96 mmol/l for IVGTT load_trend-fre-%IBW_, the highest of the 6 alternatives The equivalent figures for the IG9 set were an average change in ΔG0 of 0.21 mmol/L per unit SD for BSA and 0.92 for %IBW. IVGTT load_trend-free-BSA_ therefore generated the most generalisable trend-free variation in ΔG0 with anthropometric measures of body size.

Taking associations of ΔG0 with %IBW as an example, and translating standardised into scaled associations, in the IG16 set a unit SD change in %IBW was associated with a 0.174 mmol/L increase in ΔG0 generated by IVGTT load_trend-free-BSA_ (*Supplementary section Table 1, panel A*). One standard deviation in %IBW represented 13.2 %IBW and, In the 70-120 %IBW range in the data, the overall change in IVGTT load_trend-free-BSA_ -generated ΔG0 would, therefore, be expected to be 0.64 (=50*(0.17/13.2)) mmol/L. The observed increase in ΔG0 with 0.5g/kg IVGTT loading between 70 and 120%IBW ranged from 12.2 mmol/L (in the range 70-80%IBW) to 16.5 (in the range 110-120%IBW). Therefore, with ΔG0 generated by IVGTT load_trend-free-BSA_, the trend in ΔG0 with increasing adiposity was reduced to 15% (=(0.64/(16.5-12.2))*100) of the trend apparent with 0.5g/kg loading. The equivalent figure for the IG9 set was 16%.

*3) Generalisability across sex, adiposity and diagnoses and medications likely to affect carbohydrate metabolism*

IVGTT load_trend-free-BSA_ (generated according to the formula IVGTT load_trend-free-BSA_ = (BSA*23.9) -9.6) provided the most generalisable trend-free ΔG0. ΔG0 generated by IVGTT load_trend-free-BSA_ was, therefore, selected for evaluation of differences in the strengths of relationship of ΔG0 with %IBW according to sex, obesity, and diagnoses and medications likely to affect carbohydrate metabolism. For comparison, the same analyses were undertaken for ΔG0 generated by 0.5g/kg and by 20g/m^2^ loading and in both IG16 and 1G9 sets.

Regardless of IVGTT load formula, the rate of increase in ΔG0 with %IBW was slower in men than in women (*Supplementary section Table 2 and Figure 6*) and differences between coefficients were statistically significant in the IG16 set for ΔG0 generated by 0.5g/kg and by IVGTT load_trend-free-BSA_ loadings (both p<0.05). In the IG16 set, the rates of increase in ΔG0 were also lower among those with diagnoses likely to affect carbohydrate metabolism (0.5g/kg and IVGTT load_trend-free-BSA_ loadings both p<0.001 and 20g/m^2^ loading p<0.05) but these differences were not apparent in the IG9 set, differences between coefficients being greater in those with diagnoses and the difference significant (p<0.05) with 20g/m^2^ loading.

**Supplementary section Table 2. Regression coefficients (95%CI)^significance^ for the associations between ΔG0 and %IBW according to sex, obesity and diagnoses and medications likely to affect carbohydrate metabolism. Coefficients are for incG0 vs %IBW for: A) 0.5g/kg IVGTT glucose loading; B) 20g/m^2^ IVGTT glucose loading; and C)** IVGTT load_trend-free-BSA_**, according to the formula:** IVGTT load_trend-free-BSA_ **= (BSA*23.9) -9.6. (Significant differences between pairs of coefficients: * p<0.05, *** p<0.001).**

|  |  |  | IVGTT loading |  |
| --- | --- | --- | --- | --- |
|  | n | A) 0.5g/kg | B) 20g/m^2^ | C) BSA-derived  trend-free |
| **IG16 set (n=2,951)** |  |  |  |  |
| women | 1798 | 1.111  (0.972,1.249)^<0.001^ | 0.033  (-0.117,0.184)^0.6^ | 0.335  (0.200,0.470)^<0.001^ |
| men | 1153 | 0.873*  (0.732,1.014)^<0.001^ | -0.136  (-0.278,0.006)^0.06^ | 0.093*  (-0.040,0.226)^0.1^ |
|  |  |  |  |  |
| BMI<30kg/m^2^ | 2828 | 1.106  (0.985,1.227)^<0.001^ | -0.122  (-0.253,0.009)^0.06^ | 0.275  (0.158,0.392)^<0.001^ |
| BMI≥30kg/m^2^ | 124 | 0.886  (0.418,1.353)^<0.001^ | 0.164  (-0.262,0.589)^0.4^ | 0.264  (-0.140,0.668)^0.2^ |
|  |  |  |  |  |
| no diagnoses | 1864 | 1.263  (1.118,1.408)^<0.001^ | 0.060  (-0.099,0.218)^0.4^ | 0.433  (0.292,0.574)^<0.001^ |
| diagnoses | 1088 | 0.825***  (0.686,0.964)^<0.001^ | -0.228*  (-0.373,-0.082)^0.002^ | 0.055***  (-0.077,0.186)^0.4^ |
|  |  |  |  |  |
| no medications | 1663 | 0.911  (0.788,1.034)^<0.001^ | -0.260  (-0.394,-0.126)^<0.001^ | 0.098  (-0.021,0.216)^0.1^ |
| medications | 1289 | 0.998  (0.857,1.139)^<0.001^ | -0.135  (-0.289,0.018)^0.08^ | 0.204  (0.066,0.341)^0.004^ |
|  |  |  |  |  |
| **IG9 set (n=856)** |  |  |  |  |
| women | 759 | 0.987  (0.833,1.141)^<0.001^ | -0.066  (-0.234,0.101)^0.4^ | 0.236  (0.086,0.385)^<0.001^ |
| men | 105 | 0.663  (0.324,1.002)^<0.001^ | -0.137  (-0.478,0.204)^0.4^ | 0.026  (-0.292,0.345)^0.8^ |
|  |  |  |  |  |
| BMI<30kg/m^2^ | 738 | 1.027  (0.778,1.276)^<0.001^ | -0.120  (-0.400,0.161)^0.4^ | 0.265  (0.018,0.511)^0.03^ |
| BMI≥ BMI<30kg/m^2^ | 118 | 0.380  (-0.149,0.909)^0.1^ | -0.279  (-0.783,0.225)^0.2^ | -0.149  (-0.615,0.316)^0.5^ |
|  |  |  |  |  |
| no diagnoses | 389 | 0.751  (0.338,1.164)^<0.001^ | -0.537  (-1.017,-0.057)^0.02^ | -0.026  (-0.443,0.391)^0.9^ |
| diagnoses | 465 | 1.009  (0.815,1.203)^<0.001^ | 0.071*  (-0.134,0.276)^0.4^ | 0.301  (0.116,0.485)^0.001^ |
|  |  |  |  |  |
| no medications | 367 | 0.926  (0.746,1.106)^<0.001^ | -0.075  (-0.276,0.126)^0.4^ | 0.195  (0.018,0.371)^0.03^ |
| medications | 487 | 0.999  (0.782,1.216)^<0.001^ | -0.085  (-0.321,0.151)^0.4^ | 0.236  (0.025,0.447)^0.02^ |

**Supplementary section Figure 6.** **Regression coefficients (filled circles) and 95%CIs for the associations between ΔG0 and %IBW for (left-side and right-side respectively****): 1) women and men; 2) participants with <BMI<30kg/m^2^ and participants with BMI ≥30kg/m^2^; 3) participants with no diagnoses associated with disturbances in carbohydrate metabolism and participants with diagnoses; 4) participants taking no medications associated with disturbances in carbohydrate metabolism and participants taking medications. Coefficients are for ΔG0 vs %IBW for: A) 0.5g/kg IVGTT glucose loading; B) 20g/m^2^ IVGTT glucose loading and C)** IVGTT load_trend-free-BSA_ **loading, according to the formula:** IVGTT load_trend-free-BSA_ **= (BSA*23.9) -9.6. For reference, the coefficient for ΔG0 vs %IBW generated by** IVGTT load_trend-free-BSA_ **without separation according to the four groupings is shown (open circles).**


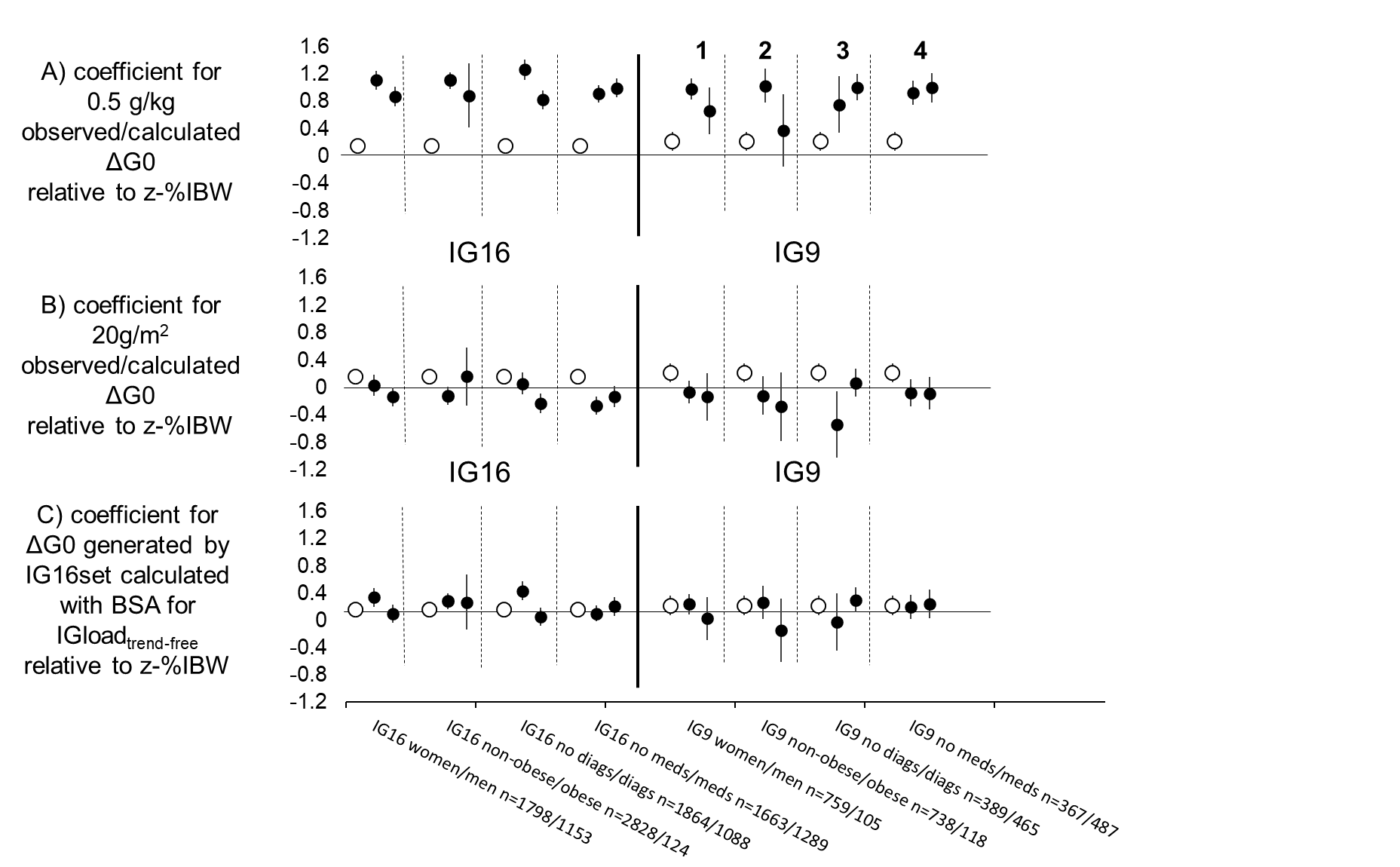

Supplement: Supplementary file 1 — Supplementary Information. [file 41598_2024_54584_MOESM1_ESM.docx]
